# Lattice site–dependent metal leaching in perovskites toward a honeycomb-like water oxidation catalyst

**DOI:** 10.1126/sciadv.abk1788

**Published:** 2021-12-10

**Authors:** Yubo Chen, Yuanmiao Sun, Maoyu Wang, Jingxian Wang, Haiyan Li, Shibo Xi, Chao Wei, Pinxian Xi, George E. Sterbinsky, John W. Freeland, Adrian C. Fisher, Joel W. Ager, Zhenxing Feng, Zhichuan J. Xu

**Affiliations:** 1 School of Material Science and Engineering, Nanyang Technological University, 50 Nanyang Avenue, Singapore 639798, Singapore.; 2 The Cambridge Centre for Advanced Research and Education in Singapore, 1 CREATE Way, Singapore 138602, Singapore.; 3 School of Chemical, Biological, and Environmental Engineering, Oregon State University, Corvallis, OR 97331, USA.; 4 Institute of Chemical and Engineering Sciences, A*STAR, 1 Pesek Road, 627833, Singapore.; 5 State Key Laboratory of Applied Organic Chemistry, Key Laboratory of Nonferrous Metal Chemistry and Resources Utilization of Gansu Province, College of Chemistry and Chemical Engineering, Lanzhou University, Lanzhou 730000, China.; 6 X-ray Science Division, Advanced Photon Source, Argonne National Laboratory, 9700 S Cass Avenue, Argonne, IL 60439, USA.; 7 Department of Chemical Engineering, University of Cambridge, Cambridge CB2 3RA, UK.; 8 Department of Materials Science and Engineering, University of California at Berkeley, Berkeley, CA 94720, USA.; 9 Berkeley Educational Alliance for Research in Singapore Ltd., 1 CREATE Way, Singapore 138602, Singapore.; 10 Energy Research Institute at Nanyang Technological University, 50 Nanyang Avenue, Singapore 639798, Singapore.

## Abstract

Metal leaching during water oxidation has been typically observed in conjunction with surface reconstruction on perovskite oxide catalysts, but the role of metal leaching at each geometric site has not been distinguished. Here, we manipulate the occurrence and process of surface reconstruction in two model ABO_3_ perovskites, i.e., SrSc_0.5_Ir_0.5_O_3_ and SrCo_0.5_Ir_0.5_O_3_, which allow us to evaluate the structure and activity evolution step by step. The occurrence and order of leaching of Sr (A-site) and Sc/Co (B-site) were controlled by tailoring the thermodynamic stability of B-site. Sr leaching from A-site mainly generates more electrochemical surface area for the reaction, and additional leaching of Sc/Co from B-site triggers the formation of a honeycomb-like IrO*_x_*H*_y_* phase with a notable increase in intrinsic activity. A thorough surface reconstruction with dual-site metal leaching induces an activity improvement by approximately two orders of magnitude, which makes the reconstructed SrCo_0.5_Ir_0.5_O_3_ among the best for water oxidation in acid.

## Teaser

Manipulating perovskite surface reconstruction offers a new path for developing advanced water oxidation catalysts.

## INTRODUCTION

Increased energy demands and global warming issues are urging our society to rely more on renewable energy sources, which are often intermittently available. To mitigate this issue, converting electrical energy (provided by renewable energy sources) into chemical bonds through electrocatalysis, such as water electrolysis for hydrogen fuel, is a viable choice for energy storage. In water electrolysis, the oxygen evolution reaction (OER) via a four-electron transfer process plays a pivotal role in determining the energy conversion efficiency. Its sluggish reaction kinetic greatly constrains the efficiency of the whole reaction, making the development of highly efficient OER catalysts one of the major challenges for implementing water electrolysis. To date, a wide variety of transition metal (TM)–based materials have been explored for catalyzing the OER in acid and alkaline, and substantial improvements have been achieved (*1*–*6*). More recently, the existence of OER-induced surface reconstruction, mainly ion leaching and/or structural reorganization, has been widely detected at various catalysts, which range from metal alloys, metal sulfides/selenides/nitrides/phosphides, and metal oxides (*4*, *7*–*13*). Among them, the perovskite-type complex oxides, such as (Ba_0.5_Sr_0.5_)(Co_0.8_Fe_0.2_)O_3-δ_ and SrIrO_3_, are demonstrated with superior activity toward OER because of the presence of unique surface reconstructions (*4*, *12*–*19*). However, although the observations of perovskite surface reconstruction have been intensively reported, designing advanced perovskite precatalysts to generate highly active reconstructed surfaces for OER is still a challenge. This, to a great extent, is due to the complexity of the entire reconstruction process, which has not been fully understood (*4*, *12*, *14*, *20*).

Specifically, in most inorganic ABO_3_ perovskites, A-site is occupied by alkaline earth metals and lanthanides, which, with their relatively large ionic size (>1 Å), are indispensable for supporting the framework of corner-shared B-site octahedra. Some A-site cations are soluble in water, even in alkaline conditions (*21*). As a result, a heavy dissolution of A-site cations is widely observed during the surface reconstruction in benchmark perovskite catalysts (*4*, *12*). The B-site can be occupied by various TMs, which are active toward catalyzing OER. Normally, TMs with good thermodynamic stability are used as B-site cations. For example, in alkaline conditions, Fe, Co, and Ni are generally used, while Ir is used in acidic conditions because of its high corrosion resistance (*4*, *13*, *22*, *23*). Thus, the leaching of B-site cations is rather weak as compared with that of A-site cations. Cation leaching is generally accompanied by a considerable increase in electrochemical surface area (ECSA) for OER (*14*, *15*). Nonetheless, a few studies have also revealed that deliberate cation leaching (as sacrificial agent) from the initial bulk can induce the formation of unique local structural environments, such as reactive surface hydroxyls and activated oxygen ligands, which can promote the activity (*9*, *24*, *25*). Given that a typical perovskite contains two types of metal cations (A-site and B-site) in totally different structural environments and both of them can leach out during the surface reconstruction, identifying the role of metal cation leaching at each site is pivotal for understanding the relationship between activity evolution and surface reconstruction. On the other hand, a consensus is that the reconstructed surface, in contact with an electrolyte, is the final active phase toward OER (*26*, *27*). Therefore, a better understanding of the formed surfaces is essential too. Until now, the formed active surface phases after metal cation leaching have been case-by-case elucidated (*4*, *12*, *13*, *15*). A resemblance of the reconstructed surface phases to the (oxy)hydroxides has been suggested on the basis of the detection of edge-shared octahedra with x-ray absorption analysis (*12*, *15*). The formation of phases, akin to the initial perovskite structure, has also been predicted by density functional theory (DFT) calculations in an SrIrO_3_ perovskite (*4*). Nevertheless, probing the exact structural motif of the surface phase is still challenging, as the perovskite surface is generally amorphized after the reconstruction.

Here, we demonstrate a step-by-step strategy to control the metal cation leaching from each geometric site in two Ir-based perovskites for understanding their activity evolution and the role of metal leaching at each site. The perovskites of SSI and SCI are used as the model catalysts. We find that metal cation leaching and the accompanying surface reconstruction can be controlled by tailoring the thermodynamic stability of B-site cations. A thorough reconstruction, including metal cation leaching and structural rearrangement, induces a remarkable activity improvement by approximately 150 times [1.5 V versus reversible hydrogen electrode (RHE)], which makes SCI among the best catalysts for OER in acid. It is found that A-site cation leaching creates more electrochemical area available for catalyzing OER, and additional B-site cation leaching induces the formation of a highly active amorphous IrO*_x_*H*_y_* surface phase, which has an intrinsic activity more than two orders of magnitude higher than the activity of a rutile IrO_2_. Surface-sensitive x-ray absorption analysis and DFT simulations indicate a honeycomb-like structure of the reconstructed amorphous IrO*_x_*H*_y_*.

## RESULTS

### Theoretical prediction of model perovskites’ surface stability

**Fig. 1.**
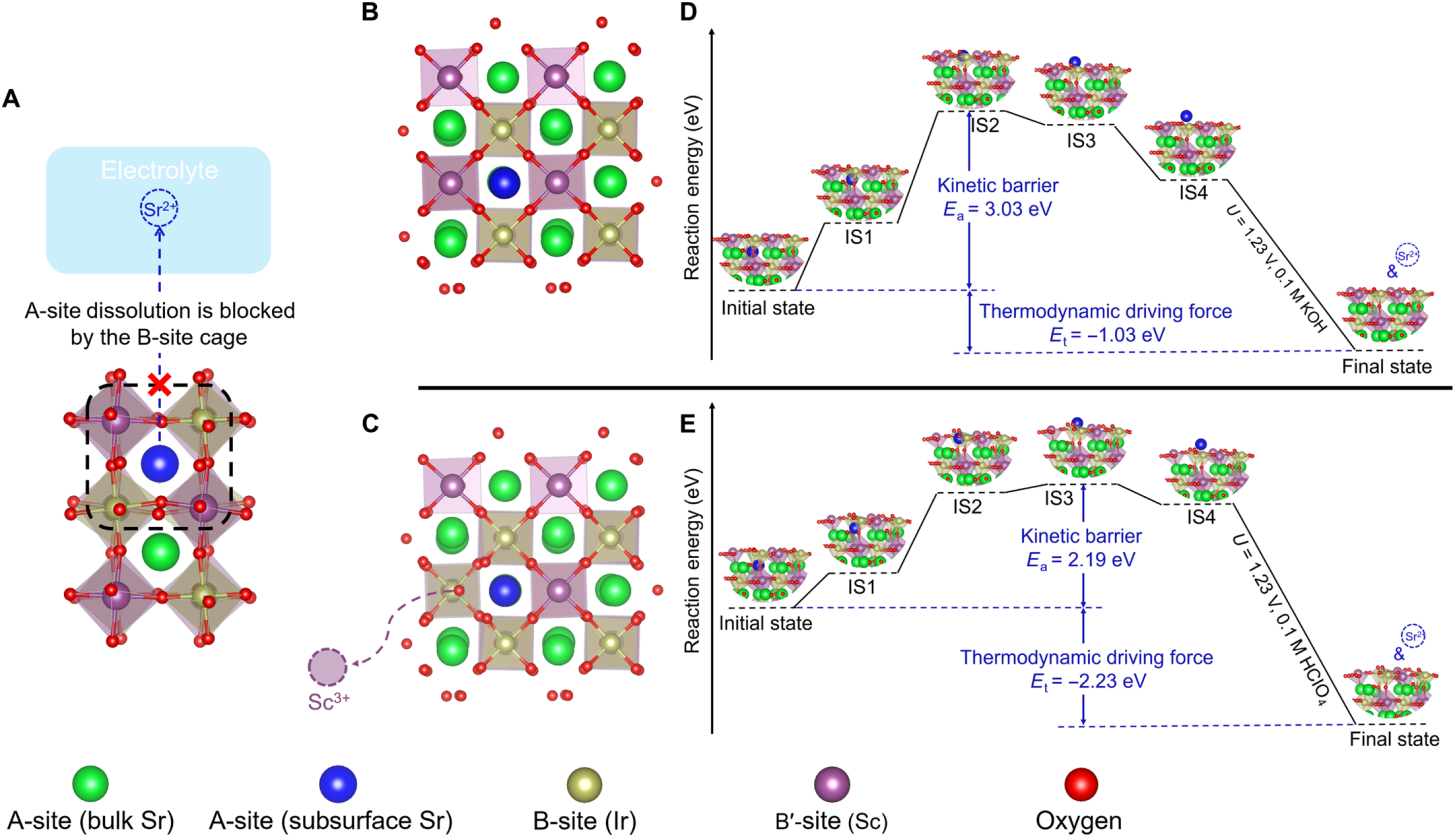
Theoretical prediction of model perovskites’ surface stability. (**A**) A schematic shows that the dissolution of A-site Sr (the blue ball), from the subsurface layer of SSI to electrolyte, can be kinetically blocked by the cage composed of B-site (Ir/Sc) octahedra. The Sr atom away from the surface is considered as bulk Sr (the green ball). (**B** and **C**) Surface of SSI without (B) and with (C) a B-site (Sc) vacancy. (**D** and **E**) Energy diagrams that illustrate the dissolution of A-site (Sr) from the subsurface of SSI without (D) and with (E) a B-site (Sc) vacancy. In (B) to (E), for a better illustration, only the selected subsurface Sr atom, which migrates to the outer surface, is marked with blue color. All the rest of subsurface Sr atoms are marked with green color.

For evaluating activity evolution during surface reconstruction and identifying the effects of metal leaching, the ability to precisely manipulate surface metal leaching is a prerequisite. Here, starting from the perovskite structure, we propose a new surface reconstruction mechanism, which allows us to precisely control perovskite surface metal leaching and accompanying reconstruction. Figure 1A shows a typical perovskite structure of SSI, in which the soluble A-site Sr atom locates in the cage composed of dense-packed B-site (Ir/Sc) octahedra. For the possible leaching of the Sr atom from the perovskite lattice to electrolyte, a large obstacle exists if the B-site octahedra are highly stable. In other words, leaching of A-site Sr and perovskite surface stability are correlated with the thermodynamic stability of B-site cations. In the model perovskites of SCI and SSI, the B-site elements of Co and Sc, whose solubility is sensitive to the pH of the electrolyte, are then used for tuning stability (fig. S1). For a better understanding of such an effect toward controlling the A-site dissolution, computational studies were performed. We postulate a new model, integrating both the lattice A-site cation migration (from the subsurface to the outer surface) and cation dissolution (from the outer surface to the electrolyte), to explain perovskite surface reconstruction at the atomic level. As illustrated in Fig. 1 (B to E) (SSI) and fig. S2 (SCI), the thermodynamic stability of B-site is simulated by constructing two perovskite surfaces without (Fig. 1B) and with (Fig. 1C) a B-site vacancy, respectively.

The dissolution of Sr includes two stages (Fig. 1, D and E). The first stage, from the initial state to intermediate state 4 (IS4), is the migration of a lattice Sr from the subsurface to the outer surface, which is controlled by a kinetic barrier. In this stage, the subsurface Sr will migrate straight toward the outer surface. This is also a likely diffusion path of A-site cation in the perovskite lattice (*28*). The intermediate states are then generated along the straightforward path. The second stage (from IS4 to the final state) is the dissolution of outer-surface Sr to Sr^2+^, which is thermodynamically favorable. The calculated free energies of different surfaces are listed in table S1. Noted that, for the second stage, the effects of both potential and pH are considered (*29*). Specifically, a potential of 1.23 V (versus RHE), which is the thermodynamic equilibrium potential of water electrolysis, is applied. Because Sc is only stable in high-pH solutions, 0.1 M KOH (pH 12.82) and 0.1 M HClO_4_ (pH 1.08) are evaluated for the creation of the surface without a B-site (Sc) vacancy and with a B-site (Sc) vacancy in SSI, respectively. More calculation details for the second stage are discussed in fig. S3.

At the ideal surface of SSI without any B-site (Sc) defects, the migration of a lattice Sr from the subsurface to the outer surface requires an activation energy (*E*_a_) of 3.03 eV (Fig. 1D), which is much higher than 2.19 eV from the surface with a B-site (Sc) vacancy (Fig. 1E). The lower *E*_a_ in the surface with a Sc vacancy can be explained by the larger space available for Sr migration (see fig. S4 with additional discussion). A similar decrease in activation energy (from 2.45 to 1.95 eV) is obtained in SCI by creating a surface B-site (Co) vacancy (fig. S2). Apparently, the migration of subsurface Sr to the outer surface becomes easier when a B-site vacancy appears. This corresponds well with the speculation that the leaching of subsurface Sr is determined by the B-site cages. Note that, in a real case, the kinetic barrier will further decrease, as multiple surface B-site vacancies can exist simultaneously because of the thermodynamically unstable feature of B-site cations. Thus, the kinetic barrier can be very sensitive to the thermodynamic stability of B-site cations. As for the second stage (IS4 to the final state), the thermodynamic driving force (*E*_t_) is always negative, indicating that the Sr dissolution can be spontaneous after it migrates to the outer surface (IS4). The computational studies suggest that, in the proposed perovskite reconstruction mechanism, the dissolution of A-site cations is strongly correlated with the stability of B-site cages.

### The initial state of Ir in model perovskite catalysts

**Fig. 2.**
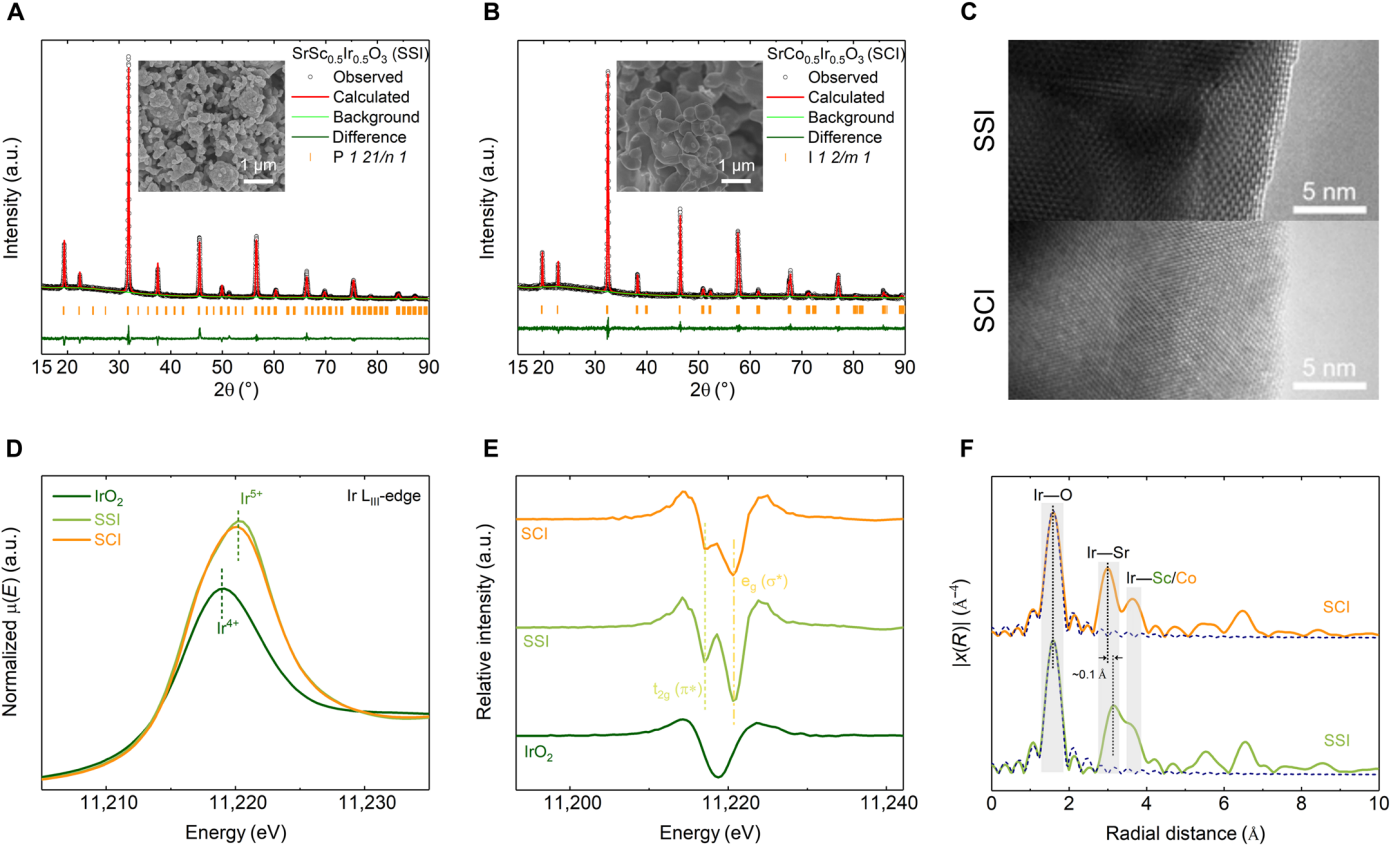
The initial state of Ir in model perovskite catalysts. (**A** and **B**) Rietveld refinement of the XRD patterns from SSI (A) and SCI (B). Insets are the corresponding scanning electron microscopy images. (**C**) High-resolution transmission electron microscopy (HR-TEM) images from the surfaces of two pristine perovskites. (**D** and **E**) XANES spectra collected at Ir L_III_-edge (D) and the corresponding second derivative (E) from SSI, SCI, and rutile IrO_2_. (**F**) Fourier-transformed *k*^3^-weighted Ir L_III_-edge EXAFS spectra from pristine SSI and SCI. The dashed lines are the fitting profiles for the first Ir─O shell. a.u., arbitrary units.

Following the theoretical prediction, the two model perovskites of SSI and SCI were synthesized via a solid-state method (see Materials and Methods for details). Their double perovskite structures, with high phase purity, are confirmed by Rietveld refinement of the x-ray powder diffraction (XRD) patterns (Fig. 2, A and B, and table S2). As shown in the insets of Fig. 2 (A and B), SSI has a relatively smaller particle size. This corresponds well with the measured Brunauer-Emmett-Teller (BET) surface areas of 0.53 and 0.18 m^2^ g^−1^ for SSI and SCI, respectively.

The local crystal structure of model perovskites was studied with high-resolution transmission electron microscopy (HR-TEM). As shown in Fig. 2C and fig. S5, the HR-TEM images of the two pristine perovskites show a highly ordered atomic arrangement. The surface regions with the perovskite-type structure were further confirmed by indexing the corresponding fast Fourier transform images with the XRD refinement results (fig. S6).

The initial states of Ir in the lattice of both perovskites were studied with x-ray absorption spectroscopy (XAS). As shown in Fig. 2D, the Ir L_III_-edge (while lines) positions of SSI and SCI are close to each other but are ~1.3 eV higher than those of Ir^4+^ in rutile IrO_2_. According to previous studies, this position shift is likely due to Ir being in a pentavalent state in SSI and SCI (*30*, *31*). By analyzing the K-edge spectra of Co and Sc (fig. S7), we confirm that both Co and Sc are in the perovskite structure. For a better understanding of the electronic structure of Ir in perovskites, the second derivative of Ir L_III_-edge spectra (white lines) of SSI and SCI is plotted in Fig. 2E. In the second-derivative curves of the two perovskites, two well-resolved peaks can be observed, while only one broad peak is found in IrO_2_. The features of the Ir L_III_-edge white lines that are related to the electric dipole allowed transition from occupied 2p states to unoccupied 5d states. Therefore, the two peaks reflect the transitions from occupied 2p states of oxygen to the unoccupied t_2g_ and e_g_ orbitals of Ir^5+^ (t_2g_^4^e_g_^0^), respectively (*31*, *32*). The observed similar characteristics of white lines of SSI and SCI that indicate the electronic structures of Ir from both model perovskites are nearly the same.

The local structure environment around Ir in the lattice was studied on the basis of the corresponding extended x-ray absorption fine structure (EXAFS) at the Ir L_III_-edge (Fig. 2F). Three typical scattering peaks, with positions of ~1.6, ~3.0, and ~3.6 Å, can be observed in the Fourier transform of the EXAFS spectra. The first peak denotes the Ir─O bond. The fitting of the first peaks (table S3) shows that Ir in both perovskites is coordinated with six neighboring O atoms, indicating that Ir is fully coordinated. The corresponding Ir─O bond lengths are 1.953 ± 0.006 Å in SSI and 1.950 ± 0.007 Å in SCI, both of which are close to the values (between 1.95 and 1.96 Å) reported for low-spin Ir^5+^ in other perovskites (*32*). The second and third peaks reflect the Ir─Sr and Ir─(Co, Sc) distance, respectively. Considering the right shift of the second peak in SSI, a slightly larger Ir─Sr distance in SSI than that in SCI is expected. This is caused by the expansion of lattice volume, which increased from 240.80 ± 0.01 Å^3^ for SCI to 254.59 ± 0.01 Å^3^ for SSI (table S2). This lattice difference is related to the larger ionic size of Sc^3+^ (0.745 Å) than that of Co^3+^ (0.545 Å for low spin and 0.61 Å for high spin). Because of the strong overlap between the second and third peaks, such distance increment is indistinguishable from the third peak in SSI. The large Ir─(Co, Sc) distances (~3.6 Å) observed in SSI and SCI confirm that the IrO_6_ octahedron is corner-shared with the neighbor (Co, Sc)O_6_ octahedron. The similar EXAFS spectra, obtained from the two model perovskites, indicate a nearly identical local structure environment for Ir. In other words, the fully coordinated Ir^5+^O_6_ octahedra are mono-μ-oxo–bridged to (Co, Sc)O_6_ octahedra, constraining Sr (A-site cation) in a B-site cage.

### Surface reconstruction in model perovskites

In previous reports, the surface reconstruction of catalysts during electrochemical cycling usually comes along with gradually increased double-layer capacitance (DLC), intensity of redox peaks, and catalytic activity (*13*, *15*, *25*, *33*). The activity improvement is generally related to increased electrochemical area and/or the formation of a more active surface phase(s) after reconstruction (*4*, *13*, *15*, *25*). To understand the surface reconstructions of model perovskites, we started with the cyclic voltammetry (CV) measurements.

**Fig. 3.**
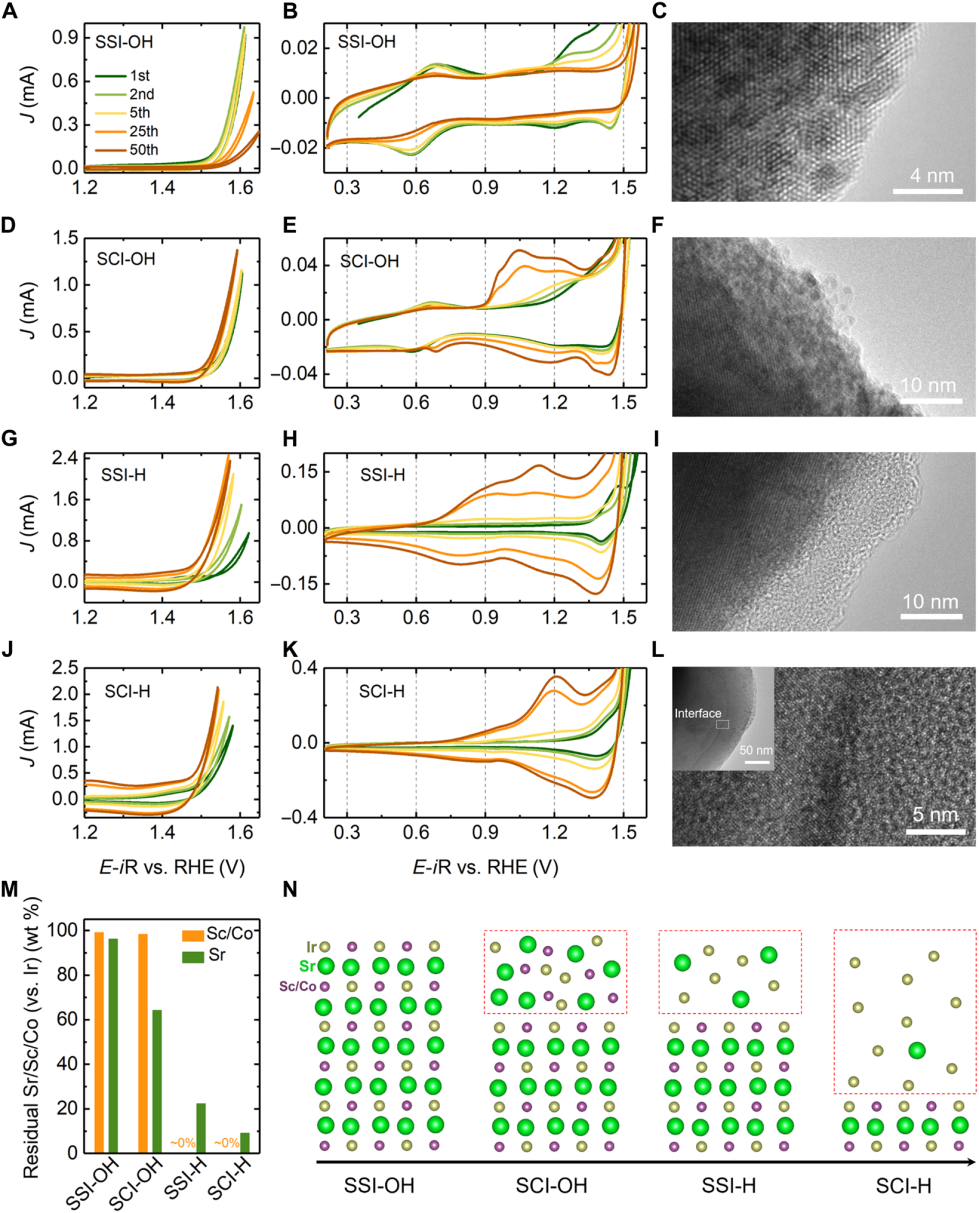
Surface reconstruction in model perovskites. (**A** to **C**) CV profiles (A and B) and surface TEM image (C) of SSI cycled in 0.1 M KOH (SSI-OH). (**D** to **F**) CV profiles (D and E) and surface TEM image (F) of SCI cycled in 0.1 M KOH (SCI-OH). (**G** to **I**) CV profiles (G and H) and surface TEM image (I) of SSI cycled in 0.1 M HClO_4_ (SSI-H). (**J** to **L**) CV profiles (J and K) and surface TEM image (L) of SCI cycled in 0.1 M HClO_4_ (SCI-H). (**M**) Surface composition change in all four samples from the XPS results. (**N**) Schematic of the surface status in all four samples.

Figure 3 summarizes the CV profiles of the model perovskites, which are cycled in either 0.1 M KOH or 0.1 M HClO_4_. As shown in Fig. 3A, the activity of SSI, cycled in an alkaline condition (SSI-OH), gradually decreases. The CV profiles (Fig. 3B) show an apparent redox peak at ~0.6 V versus RHE, which should be related to the Ir^3+^/Ir^4+^ redox transition (*34*). Only the Ir in the outer surface contributes to this redox transition. This is supported by the integral charge of the cathodic peak at ~0.6 V of the first CV cycle that is close to the charge (Ir^4+^ to Ir^3+^) estimated by assuming that only the lattice Ir from the outer surface of SSI is involved. More details are shown in fig. S8. Additional redox peaks of more than ~1.2 V versus RHE are also observed. These peaks can be related to the redox transition of Ir^4+^/Ir^5+^, which occurs at high overpotentials (*35*, *36*). During the cycling tests, intensities of the redox peaks and the OER current gradually decrease. In SSI, Sr^2+^ and Sc^3+^ have fixed oxidation states and therefore have no contribution to the redox peaks. Thus, the gradual disappearance of redox peaks in the CV indicates the loss of surface-active sites (Ir) in SSI. Although Ir can be one of the most stable elements, it has been proven that the highly oxidized Ir^6+^ (${\text{IrO}}_{4}^{2-}$) can be soluble (*37*). Thus, the Ir in the outermost surface, which contributes to the measured OER catalytic activity, can dissolve during the cycling. Meanwhile, the DLC remains unchanged during the cycling, hinting that the surface of SSI can still be highly stable. According to the HR-TEM image of the SSI after the cycling (Fig. 3C and figs. S5 and S6), the surface of SSI-OH is well crystallized. Note that we observed that some Ir nanoparticles evolved when performing TEM analysis. This is probably due to the electron beam illumination, and the same phenomenon is also observed in SCI samples (fig. S9) (*13*, *38*). The stable surface of SSI-OH is further supported by x-ray photoelectron spectroscopy (XPS) results (fig. S10 and table S4), in which the spectra of Sr_3d, Sc_2p, and Ir_4f from SSI-OH are close to the ones from pristine SSI. Therefore, the SSI cycled in alkaline conditions exhibits a stable surface structure. This corresponds well with the DFT prediction that the thermally unstable Sr is constrained in the SSI lattice in the alkaline condition.

Figure 3 (D and E) shows the CVs of SCI in an alkaline condition (SCI-OH). The activity slowly increases in the initial 25 cycles and remains constant in the following cycles. The profile of CV in the initial five cycles resembles the case of SSI-OH. However, in the 25th CV cycle, several distinctive oxidation peaks between 0.9 and 1.3 V are observed, and the peaks become more pronounced in the 50th CV cycle. Because of the existence of the Co^3/4+^ redox transition, it is difficult to distinguish the contribution of Ir and Co to the redox peaks. Nevertheless, the irreversible redox features and gradually increased DLC during cycling hint that surface reconstruction occurs during cycling. From the corresponding HR-TEM images (Fig. 3F and figs. S5 and S6), the surface of SCI-OH loses the long-range ordered perovskite structure and is amorphous with a depth of approximately 10 nm. The analysis of XPS spectra (Sr_3d, Co_2p, and Ir_4f in fig. S11 and table S5) also confirms the surface reconstruction. With Ir as a reference, ~35% Sr has dissolved from the amorphous surface. Considering the observed stable SSI-OH surface, the reconstruction of the SCI-OH surface is switched on by replacing B-site Sc with thermodynamically unstable Co. The reconstruction process can be explained as follows: (i) Slight leaching of B-site Co triggers the massive leaching of Sr, (ii) the perovskite structure cannot sustain such a high degree of A-site deficiency, and (iii) the surface loses the long-range ordering and becomes amorphous.

In low-pH value solutions, Sc is thermodynamically unstable (fig. S1). The surface of SSI is expected to be unstable in acidic conditions. Figure 3G shows the CVs of SSI measured in 0.1 M HClO_4_ (SSI-H). The activity of SSI-H steeply increases in the initial five cycles, indicating that the surface of SSI-H experiences reconstruction. As shown in Fig. 3H, a distinctive oxidation peak at ~1.45 V can be observed in the first cycle. This peak, however, disappears from the second cycle. The irreversibility and high intensity indicate that this oxidation peak is related to the fast dissolution of cations. In the following cycles, apparent increases of the redox peaks’ intensity and DLC are observed, indicating that more redox-active sites are available. From the HR-TEM image of SSI-H (Fig. 3I and figs. S5 and S6), an amorphous surface region, with a depth of ~10 nm, is observed. However, in contrast to the amorphous surface observed from SCI-OH, the XPS analysis confirms that a larger amount of Sr [~80 weight % (wt %)] and almost all Sc have been leached from the surface of SSI-H (fig. S10), which leads to the formation of an Ir-rich amorphous surface.

The CV profiles of SCI cycled in acid (SCI-H) are shown in Fig. 3 (J and K). Similar to the case of SSI-H, a gradually increased activity is observed in the initial 25 cycles. The steeply increased DLC and intensity of redox peaks highlight a heavy surface reconstruction. After cycling, the profile of the final CV resembles the one of SSI-H, indicating that a similar reconstructed surface may form over SCI-H. This is reasonable, given that the initial state of Ir in SSI and SCI is nearly identical to each other. The corresponding TEM images (Fig. 3L and figs. S5 and S6) show that the surface of SCI-H is also amorphous after the surface reconstruction. The depth of the amorphous region can reach ~50 nm in 50 CV cycles, which is much deeper than those observed in SCI-OH and SSI-H. This conspicuous surface reconstruction can be correlated with the heavy cation (Sr and Co) leaching, as evidenced by the absence of XPS signals of Sr_3d and Co_2p in SCI-H (fig. S11).

The surface composition changes of all four samples are summarized in Fig. 3M, and a schematic illustration (Fig. 3N) presents the surface status in all four cases. The first schematic shows that the surface of SSI-OH maintains the perovskite structure. The second one illustrates that the surface of SCI-OH is amorphous, but the leaching is relatively light, and A-site Sr cations are partially leached out. The third one shows that the surface of SSI-H is amorphous and the surface region is Ir-rich. A high proportion of A-site Sr and almost all B-site Sc are leached out. The last one shows the case of SCI-H, where most Sr and almost all B-site Co have been leached out from the surface. The measured activity of SSI-OH is mainly contributed by the Ir in the perovskite lattice, but the activities measured in the other three cases are most likely contributed by the Ir from the reconstructed perovskite surfaces.

### Activity evolution during surface reconstruction

**Fig. 4.**
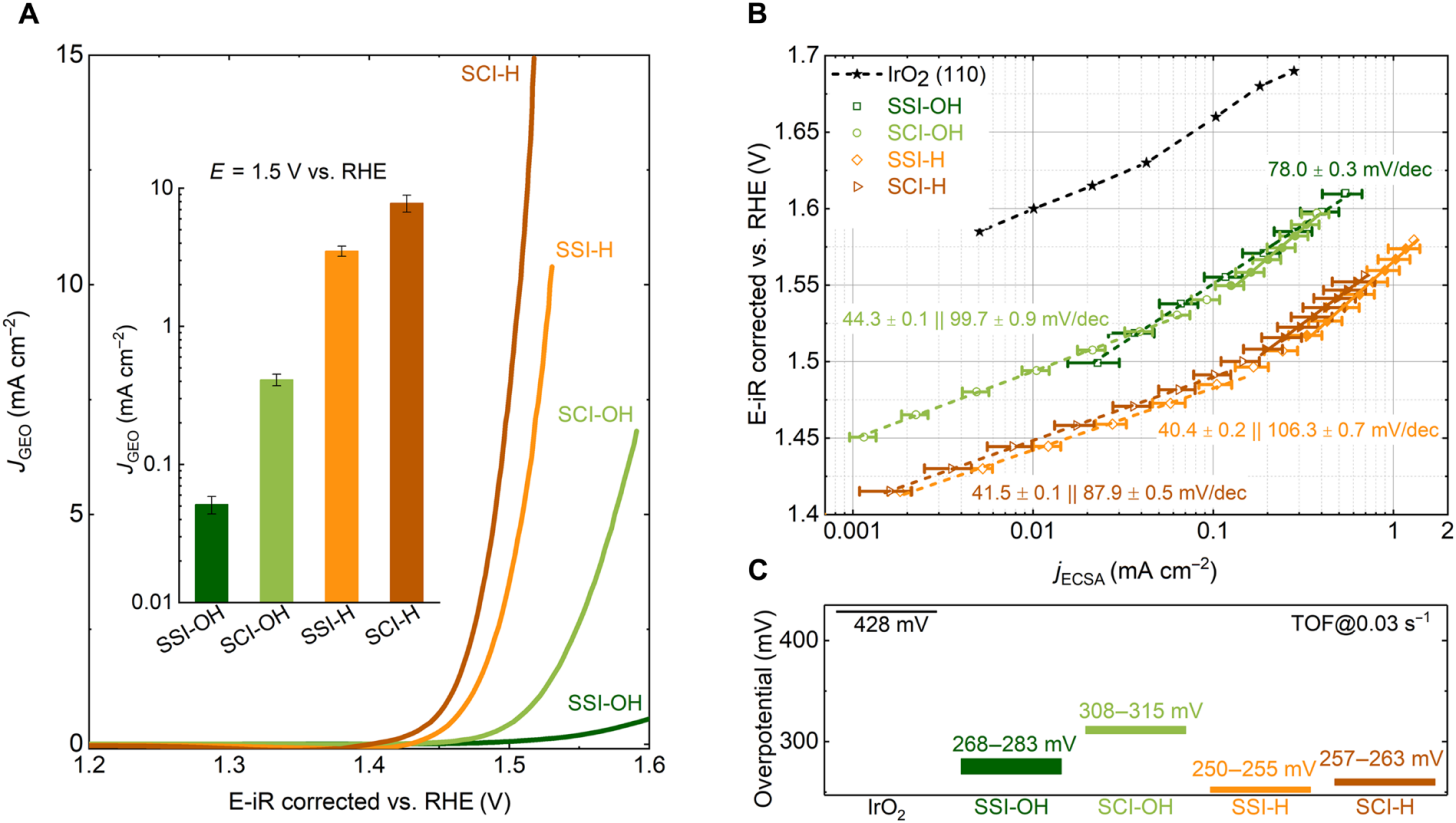
Activity evolution during surface reconstruction. (**A**) BET-normalized activities from SSI-OH, SCI-OH, SSI-H, and SCI-H. The inset shows the BET-normalized OER currents at 1.5 V versus RHE. (**B**) Intrinsic OER current (normalized to ECSA) densities versus potential from all four samples. The intrinsic OER current for IrO_2_ is from an IrO_2_ (110) thin film in 0.1 M HClO_4_ (*43*). (**C**) Overpotentials required for different samples to reach a TOF of 0.03 s^−1^. The error bars in the inset of (A) denote the SE of three independent tests.

To highlight the promoting effect of surface reconstruction for OER, the geometry surface area–normalized (GEO-normalized) activities of SSI-OH, SCI-OH, SSI-H, and SCI-H are shown in Fig. 4A. The inset shows the GEO-normalized OER currents at 1.5 V versus RHE. Although the initial perovskite structures of four samples are similar, the GEO-normalized activities of SCI-OH, SSI-H, and SCI-H, which undergo surface reconstruction under OER, greatly outperform the activity of SSI-OH. Specifically, at 1.5 V versus RHE, the GEO-normalized OER current of SCI-H is approximately 150 times higher than that of SSI-OH. The activity improvement is likely caused by two features during surface reconstruction. The first is the reconstruction of the perovskite surface from crystalline (SSI-OH) to amorphous (SCI-OH) with A-site cation (Sr) leaching. This reconstruction induces an activity improvement of approximately one order of magnitude. The second is the additional leaching of B-site cations, which induces further activity improvement (SCI-OH versus SCI-H) of approximately one order of magnitude. The surface reconstruction of perovskites, including both A-site and B-site cation leaching, is key for the measured high activity. Nonetheless, in cases where surface reconstruction occurs, the GEO-normalized activity may not precisely reflect the intrinsic activity of the reconstructed surfaces. This is because the real active area may increase during the surface reconstruction, as reflected by the steeply increased DLC during electrochemical cycling (Fig. 3). In this case, the current density normalized by ECSA is more suitable to represent the intrinsic activity of the model perovskites (*39*, *40*).

The ECSA is estimated with advanced impedance spectrum analysis (*2*, *41*, *42*). More details are discussed in figs. S12 and S13. In Fig. 4B, the intrinsic OER current densities versus potential are plotted, and the intrinsic OER current density from an IrO_2_ (110) film is also plotted for comparison (*43*). The intrinsic activity of SSI-OH is much closer to the reported OER activity of an SrIrO_3_ perovskite film with a stable surface in alkaline, confirming that the measured activity originates from Ir in the perovskite lattice (*34*). On the other hand, the intrinsic current of SCI-OH is close to the one of SSI-OH without surface reconstruction, suggesting that the initial surface reconstruction with only A-site Sr leaching contributes little to the intrinsic activity improvement. This surface reconstruction, however, generates more electrochemical areas available for OER, which explains the measured higher GEO-normalized activity of SCI-OH (Fig. 4A). More than one order of magnitude improvement in intrinsic activity is observed in SSI-H and SCI-H, which both experience additional B-site cation leaching. The steeply increased intrinsic activity indicates that certain highly active Ir sites are formed in the thoroughly reconstructed surfaces (SSI-H and SCI-H after 50 CV cycles). All Tafel plots from the three surface-reconstructed catalysts (SCI-OH, SSI-H, and SCI-H) show prominent “bending” behavior with a change in Tafel slope from ~40 mV/decade (dec) at low potentials to ~100 mV/dec at high potentials. Specifically, the Tafel plot of SCI-OH shows a bending at a potential of ~1.53 V, while the bending of Tafel plots from SSI-H and SCI-H starts at a lower potential of ~1.49 V. More recently, a universal linear relationship between log(current) and pseudocapacitive charge storage (catalyst deprotonation with hole formation) has been demonstrated at rutile IrO_2_. The observed bending of Tafel plot at ~1.58 V is related to the change in response of IrO_2_ surface hole coverages to the potential applied (*44*). With pulse voltammetry tests, we found that the logarithm of OER current from SCI-OH and SCI-H is also proportional to the corresponding charge stored during OER (figs. S14 and S15). The observed lower bending potential (less than 1.50 V) indicates that the deprotonation behavior over fully reconstructed perovskite surface is different from the case of rutile IrO_2_, hinting the unique local environment of active Ir sites in the reconstructed perovskite surfaces.

To further confirm that the high intrinsic activity is related to the formation of superior Ir sites, the turnover frequencies (TOFs) of Ir from different surfaces are calculated by taking structural effects (the arrangement of surface Ir atoms) into account. On the basis of well-defined surfaces (see Materials and Methods for details), the overpotentials required to reach a TOF of 0.03 s^−1^ are compared in Fig. 4C. The overpotential required for SSI-H and SCI-H is between 250 and 263 mV, and it is ~170 and ~60 mV lower than IrO_2_ (428 mV) and SCI-OH (308 to 315 mV), respectively. This overpotential reduction corresponds well with the measured increment of the intrinsic activity during the surface reconstruction process (Fig. 4B), confirming that the observed high intrinsic activity is due to the formation of highly active Ir. Moreover, the above activity evaluation also reveals that the leaching of B-site cations is key to the formation of highly active Ir in the reconstructed perovskite surface. That is, the highly active Ir in the reconstructed perovskite surface can only be activated when the B-site cations (Sc/Co), those adjacent to the Ir, are leached out.

### The state of active Ir site in the reconstructed surface

**Fig. 5.**
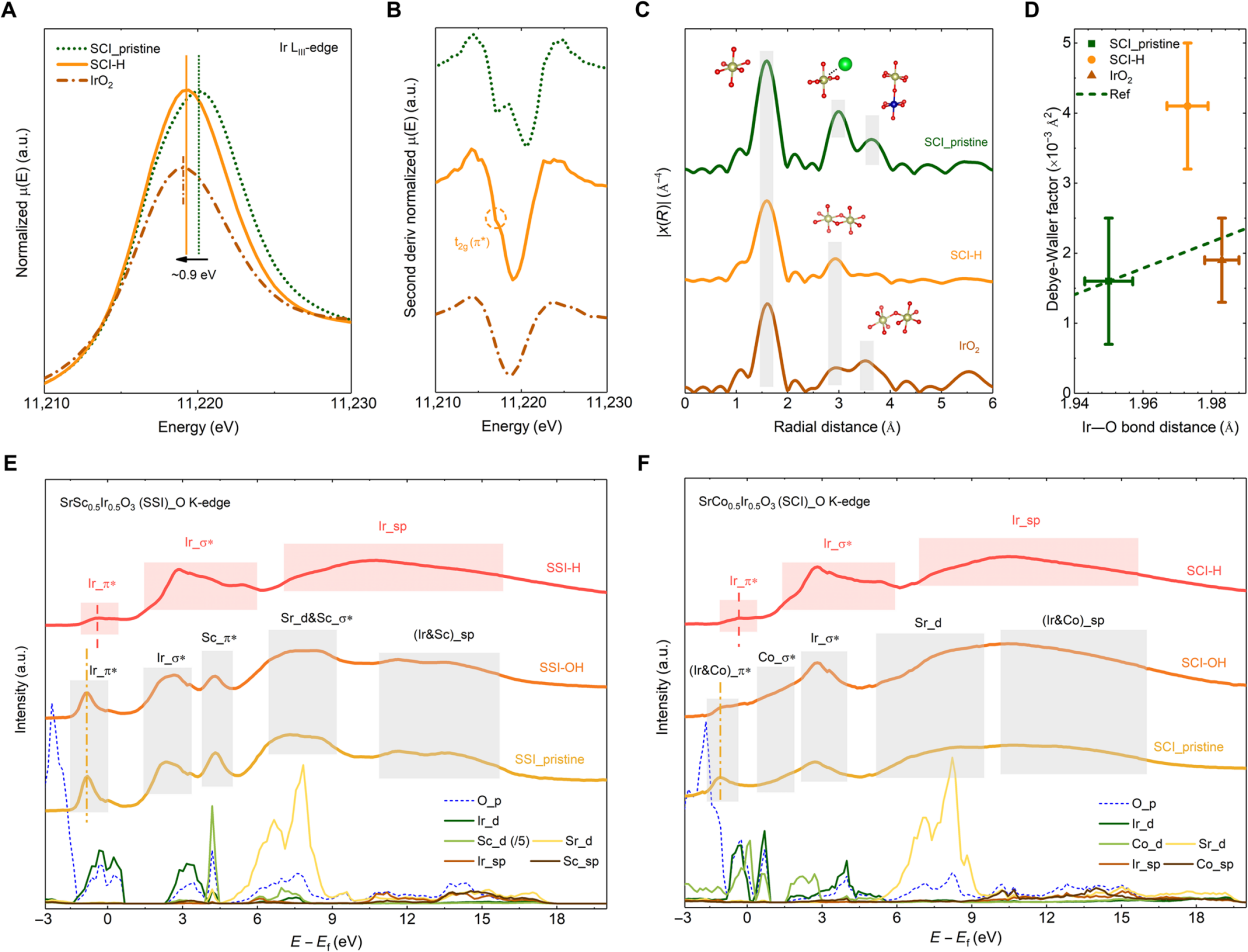
The state of the active Ir site in the reconstructed surface. (**A** and **B**) XANES spectra collected at Ir L_III_-edges (A) and the corresponding second derivative (B) from pristine SCI, SCI-H, and IrO_2_. (**C**) Fourier-transformed *k*^3^-weighted Ir L_III_-edge EXAFS spectra for pristine SCI, SCI-H, and IrO_2_. (**D**) Relationship between Ir─O bond lengths and DW factors. The dashed line is the reported positive correlation between Ir─O bond lengths and DW factors in Ir-based perovskites (*32*). (**E** and **F**) O K-edge spectra from the pristine and electrochemically cycled SSI (E) and SCI (F). Projected density of states (PDOS) of the O_p, Ir_d, Sc_d, Co_d, Sr_d, Ir_sp, Sc_sp, and Co_sp state from pristine SSI (E) and SCI (F) that are also presented from indexing the O K-edge spectra. The intensity of the Sc_d state in SSI is divided by 5. In this figure, all the spectra are recorded in the TEY mode.

To better understand the structure of the reconstructed surface, especially the formed highly active Ir, the local structure and oxidation state of Ir in the reconstructed surface region were characterized by XAS using total electron yield (TEY) detection. SCI-H, which has a fully reconstructed surface, is used as a model sample. Figure 5A shows the Ir L_III_-edge spectrum of SCI-H. The Ir L_III_-edge spectra should originate from the highly active Ir sites in the reconstructed surface. This is because XAS in the TEY mode is more surface sensitive due to the short escape depth of electrons. The Ir L_III_-edge spectra of pristine SCI and IrO_2_ are also presented. As compared with the white line position of Ir^5+^ in pristine SCI, the white line position of Ir L_III_-edge spectra from the reconstructed surface region shifts left and is close to the white line position of Ir^4+^ in IrO_2_, indicating the tetravalent state of Ir in the reconstructed surface. The corresponding energy shift is ~0.9 eV, which is close to the reported energy shift of 0.8 to 1 eV for a unit change in the Ir oxidation state (*9*, *45*). Figure 5B shows the second derivatives of Ir L_III_-edge spectra. As compared with the pristine SCI, the second derivative of Ir L_III_-edge spectra from the reconstructed perovskite surface shows a weak peak splitting because of the splitting of d orbitals. Besides, the relative intensity of the peak, which is related to the transition to the t_2g_ orbital, becomes much lower. This should be caused by the reduction of initial Ir^5+^ (t_2g_^4^e_g_^0^) to Ir^4+^ (t_2g_^5^e_g_^0^), which has almost fully filled t_2g_ orbital after surface reconstruction.

The local structural environment of Ir in the reconstructed SCI-H surface was then studied by EXAFS. Figure 5C shows the Fourier-transformed *k*^3^-weighted Ir L_III_-edge EXAFS of pristine SCI, rutile IrO_2_, and SCI-H. From the spectrum of SCI-H, the two peaks of Ir─Sr (~3.0 Å) and Ir─Co (~3.6 Å) bonds in the perovskite structure disappear, indicating that the initial perovskite structure no longer exists in the SCI-H surface region. Instead, a new peak with a reduced distance of ~2.9 Å appears. Compared with the spectrum of rutile IrO_2_, this peak is caused by the di-μ-oxo–bridged IrO_6_ octahedra. That is, although the reconstructed SCI surface is amorphous, a large amount of edge-sharing IrO_6_ octahedra appear after the surface reconstruction. On the other hand, the typical peak reflecting corner-shared IrO_6_ octahedra in rutile IrO_2_ does not appear in the spectrum of SCI-H. The additional fitting of the first peak revealed that the Ir center in the reconstructed SCI surface is fully coordinated with six oxygen atoms (table S3). However, because of the reduction of Ir^5+^ after surface reconstruction, the average Ir─O bond length increases to 1.973 Å, which is higher than 1.950 Å in pristine SCI but comparable to that in rutile IrO_2_ (1.983 Å). In addition to the bond length, the Debye-Waller (DW) factor, which corresponds to the mean square displacement of the Ir─O bond length due to vibration and/or static disorder, can be obtained from the fitting (*32*). For the vibration, a longer Ir─O bond length with stronger thermal vibration should induce a larger DW factor. As a result, a positive correlation between Ir─O bond length and DW factor has been found in Ir-based perovskites (dashed line in Fig. 5D) (*32*). For the static disorder, both local structural defects (coordinatively unsaturated sites) and multiple bond lengths (highly distorted IrO_6_ octahedra) can also induce a high DW factor. As shown in Fig. 5D, the Ir─O bond lengths and DW factors of pristine SCI and rutile IrO_2_ are in accordance with the reported positive correlation. Nevertheless, a much higher DW factor is estimated from the fitting results of the reconstructed SCI-H surface. Considering that the Ir in the reconstructed surface is fully coordinated, the large DW factor reveals that the IrO_6_ octahedra in the reconstructed SCI surface are highly distorted. These multiple Ir─O bond lengths can be explained by the fact that Ir should bond with O, OH, and even OH_2_ after surface reconstruction (*9*).

To better assess the effect of surface reconstruction on the local electronic state of Ir, we performed soft XAS (in the TEY mode) characterization at the O K-edge. Note that, because of the low energy of soft x-ray, the probing depth of soft x-ray in the TEY mode is around a couple of nanometers (*46*). This makes the O K-edge spectrum highly sensitive to the surface. Because the unoccupied oxygen 2p band hybridizes with the unoccupied metal bands, the O K-edge spectrum can reflect the surface electronic structure changes before and after reconstruction. Figure 5 (E and F) shows the O K-edge spectra of the pristine and electrochemically cycled perovskites. The O K-edge spectra of the pristine perovskites can be well indexed with the calculated electronic structures (Ir_d and O_p). Additional details of the calculated electronic structures are shown in fig. S16. Briefly, the broad shoulders above ~5 eV are related to the hybridization of O_p, Sr_d (A-site), and Ir/Co/Sc_sp (B-site). The featured pre-edge peaks correspond to the O_p states hybridizing with t_2g_ (π*) and e_g_ (σ*) states of the B-site cations (Ir, Co, and Sc). Moreover, the O K-edge spectra of the surfaces of pristine perovskites (Fig. 5, E and F) also resemble the spectra of the corresponding bulk materials (fig. S17), confirming the perovskite structures of the initially crystallized surfaces. In Fig. 5E, the spectra of the pristine SSI and SSI-OH are almost identical to each other, confirming that the surface of SSI is highly stable when cycled in alkaline. Unlike SSI-OH, the O K-edge spectrum of SCI-OH changes, indicating that surface reconstruction occurs in alkaline (Fig. 5F). Nevertheless, all the features related to pristine perovskite SSI and SCI disappear in the spectra of SSI-H and SCI-H. Similar changes can also be observed in the O K-edge spectra of the scanning TEM–electron energy-loss spectroscopy (STEM-EELS) analysis (fig. S18). The disappearance of these features indicates that thoroughly reconstructed surfaces are formed after cycling in acid.

The observed evolution of O K-edge spectra corresponds well with the detected surface reconstructions in SSI and SCI (Fig. 3). Both O K-edge spectra of the reconstructed surfaces of SSI-H and SCI-H resemble each other (fig. S19), hinting that the two reconstructed surfaces have a nearly identical electronic structure. Thus, the active sites (local domains with short-range order) in the reconstructed amorphous perovskite surfaces can be akin to a certain IrO*_x_*H*_y_* phase with a well-defined crystal structure. In addition, the measured O K-edge spectra can be considered as a fingerprint for identifying the possible structure.

### Likely structure of the reconstructed perovskite surface

**Fig. 6.**
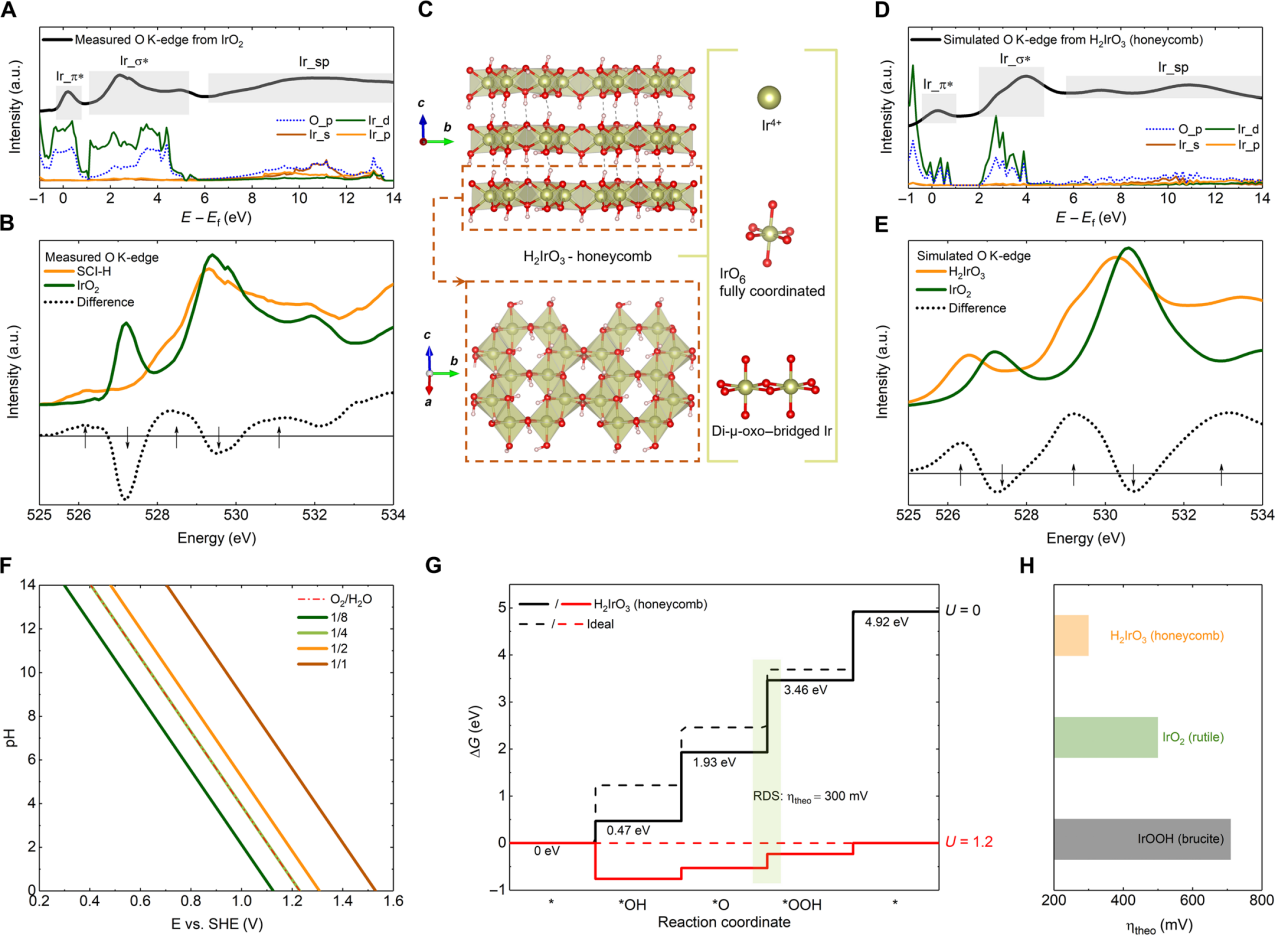
Likely structure of the reconstructed perovskite surface. (**A**) Measured O K-edge spectra, recorded in the TEY mode, and PDOS from rutile IrO_2_. The Fermi energy is set to zero. (**B**) Featured pre-edge peaks from the measured O K-edge spectra of SCI-H and IrO_2_. The dashed curve is the difference between the two measured O K-edge spectra. (**C**) An H_2_IrO_3_, with layered honeycomb structure, is the most likely structure of the reconstructed perovskite surface. (**D**) Simulated O K-edge spectra and PDOS from H_2_IrO_3_ (honeycomb). The Fermi energy is set to zero. (**E**) Featured pre-edge peaks from the simulated O K-edge spectra of H_2_IrO_3_ (honeycomb) and IrO_2_. The dashed curve is the difference between the two simulated O K-edge spectra. (**F**) Simulated pH potential phase diagram for the honeycomb H_2_IrO_3_ surface. (**G**) Standard free-energy diagram for OER. The asterisk represents the active site. SHE, standard hydrogen electrode. (**H**) Calculated theoretical overpotentials for IrOOH (brucite), IrO_2_ (rutile), and H_2_IrO_3_ (honeycomb).

To explore the most likely structure of the IrO*_x_*H*_y_* phase in the reconstructed perovskite surface, the O K-edge spectrum of rutile IrO_2_ was measured and compared with that of SCI-H with a reconstructed surface. As shown in Fig. 6A and fig. S20, the O K-edge spectra of rutile IrO_2_ have three parts, which are related to the hybridization of O_p with Ir_d (π*), Ir_d (σ*), and Ir_sp, respectively. The featured pre-edge peaks of the two O K-edge spectra and the corresponding difference are shown in Fig. 6B. Compared to the O K-edge spectra of IrO_2_, in the spectrum of SCI-H, the Ir (π*) peak is flatter and shifts ~1 eV to lower energy, while the Ir (σ*) peak resembles that of IrO_2_. On the basis of the results of XAS analysis (Fig. 5, A to D), we propose a series of possible Ir-based oxides (fig. S21), whose O K-edge spectra were simulated with the consideration of the core-hole effect (fig. S22).

As displayed in Fig. 6C, the states of Ir and O in H_2_IrO_3_ with layered honeycomb structure match well with the characteristics of the reconstructed perovskite surface. Specifically, in this honeycomb structure, the Ir^4+^ ions are fully coordinated with six oxygen atoms, and the IrO_6_ octahedra are strictly edge-sharing. The simulated O K-edge spectrum of the honeycomb structure and the corresponding density of states are presented in Fig. 6D. Three parts, corresponding to the contributions from Ir_d (π*), Ir_d (σ*), and Ir_sp, can be identified from the spectrum. The pre-edge peaks in this spectrum are compared with the simulated pre-edge peaks of rutile IrO_2_ (Fig. 6E). The difference between the simulated O K-edge spectra from H_2_IrO_3_ and IrO_2_ resembles the measured difference shown in Fig. 6B, indicating that the structure of the active site in the amorphous perovskite surface is close to this simulated honeycomb structure.

Note that, if ignoring the honeycomb character, the structure of this H_2_IrO_3_ is similar to that of transition metal (oxy)hydroxides, which are also popular catalysts for OER (*6*, *47*, *48*). In addition, the formation of certain (oxy)hydroxide(s) that feature edge-sharing octahedra has also been considered as the real active phase(s) of some highly active complex oxides with surface reconstruction (*10*–*12*). On the other hand, a layered IrOOH has also been synthesized for catalyzing OER, but the activity of this IrOOH is reported to be inferior to the rutile IrO_2_ (*49*). Considering that the intrinsic activity of the Ir (oxy)hydroxide(s) in the amorphous perovskite surface is superior to both rutile IrO_2_ and perovskite SSI (Fig. 4, B and C), the identified honeycomb structure should be the intrinsic reason for the high activity. We then performed complementary DFT calculations to explore the surface properties of H_2_IrO_3_ (honeycomb).

Considering the unusual Tafel plot bending (Fig. 4B and fig. S15), we first simulated the surface deprotonation behavior versus potential in the honeycomb H_2_IrO_3_ (Fig. 6F and fig. S23). As compared with the reported case over rutile IrO_2_ surface, desorption of protons in the honeycomb H_2_IrO_3_ is much more sensitive to the potential applied. For instance, ^1^/_2_ surface protons are ready to desorb at a potential of 1.308 V (versus RHE), while a potential of ~1.45 V is expected for rutile IrO_2_ (*44*). Then, the lower potential of Tafel plot bending in the honeycomb H_2_IrO_3_ can be ascribed to the corresponding unusual surface deprotonation behavior. In addition, the OER free energy diagrams were also computed to investigate the thermodynamic features of H_2_IrO_3_. As shown in Fig. 6G, the rate-determining step of OER on H_2_IrO_3_ is the elementary step to oxidize the *O to *OOH state, which requires a potential of 1.53 V (versus RHE) to initiate the reaction. Note that the free energy of *OH and *OOH fits well with the established scaling relation in perovskite and rutile, further supporting that the reconstructed honeycomb structure is strictly composed of edge-sharing IrO_6_ structural units (*50*). Figure 6H compares the computed reaction overpotential of IrO_2_, IrOOH, and H_2_IrO_3_. In accordance with the reported experimental result (*49*), the layered IrOOH has an even higher overpotential than rutile IrO_2_, suggesting that the reconstructed surface can hardly be in an intact and layered Ir (oxy)hydroxide phase. In contrast, the honeycomb H_2_IrO_3_ shows a much lower overpotential than IrO_2_, further supporting the high likelihood of its role in contributing to the high activity of the reconstructed surface.

## DISCUSSION

In this work, we study the surface reconstruction of two Ir-based model perovskites and the corresponding activity evolution step by step. We demonstrate a new perovskite surface reconstruction mechanism, in which the thermodynamic stability of B-site cations governs the surface stability of perovskites during OER. By tuning the B-site compositions, we successfully control the surface reconstruction in the model Ir-based perovskites of SSI and SCI. From alkaline to acid, the whole perovskite reconstruction process is divided into two for better investigation of the roles of A-site and B-site metal leaching in surface reconstruction.

We find that the reconstruction-induced activity improvement is due to two factors. First, the surface reconstruction with A-site metal cation leaching makes more electrochemical area available for OER. The second is the formation of a highly active IrO*_x_*H*_y_* phase in thoroughly reconstructed surfaces with mixed A-site and B-site metal cation leaching, and the B-site cation leaching is pivotal to the formation of such an active phase. Subsequently, with surface-sensitive O K-edge spectra as fingerprints, we identify that the active phase has a key honeycomb-like structure, which is responsible for the high activity. The activity of SCI-H after surface reconstruction is among the best toward water oxidation in acid. Given that surface reconstruction with ion leaching has been intensively observed in the catalysts for electrocatalysis, we believe that this step-by-step leaching strategy can be extended to other complex catalysts for investigating the roles of element leaching in surface reconstruction processes for better catalyst design.

## MATERIALS AND METHODS

### Synthesis of model perovskites

Both SCI and SSI were synthesized with the solid-state reaction. Stoichiometric amounts of SrCO_3_ (99.9%; Sigma-Aldrich), IrO_2_ (99.9%; Sigma-Aldrich), Co_3_O_4_ (Sigma-Aldrich), and Sc_2_O_3_ (99.9%; Sigma-Aldrich) were thoroughly ground and calcined at 1150°C (for SCI) or 1350°C (for SSI) for 12 hours under ambient air.

### Electrode preparation and electrochemical characterization

The electrodes were prepared by drop-casting as-prepared catalyst ink on a glassy carbon rotating electrode with a diameter of 5 mm (Pine Research Instrumentation). The catalyst loading was fixed at 0.05 mg. Specifically, the ink was prepared by mixing 2.5 mg of catalyst powder with 1 mg of acetylene black carbon, which was ultrasonically dispersed in the solution that contains 375 μl of H_2_O, 112.5 μl of isopropanol, and 12.5 μl of Nafion solution (5 wt %; Sigma-Aldrich). Ten microliters of well-dispersed ink was drop-casted onto the polished glassy carbon electrode, which was dried under ambient air until a robust catalyst layer formed. Noted that, for evaluating the intrinsic activity (normalized to ECSA) of different samples and for estimating charge storage with pulse voltammetry, the catalyst inks were prepared without acetylene black carbon. The electrochemical tests were conducted in either 0.1 M KOH or 0.1 M HClO_4_. OER measurements were performed with a biologic SP-150 potentiostat coupled with a modulated speed rotator (Pine Research Instrumentation). The glassy carbon electrode was used as the working electrode, a Pt wire was used as the counter electrode, and a saturated calomel electrode (SCE) was used as the reference electrode. The rotation speed for all tests was fixed at 1600 rpm. At least three measurements were performed when evaluating the OER activities of different catalysts. The pulse voltammetry was performed in the same electrochemical setup used for activity evaluation. Before pulse voltammetry tests, the electrodes were pretreated for 50 CV cycles (between 0.3 and 1.8 V versus RHE without *i*R correction) to ensure that the reconstructed surfaces reach the steady status. In pulse voltammetry, a low potential of 1.35 V, below the onset of OER, was selected (*44*), and the high potential changed from 1.42 to 1.8 V with a step of 20 mV. For both anodic and cathodic sections, the duration was fixed at 10 s and the current was recorded every 0.001 s.

### Characterization

XRD measurements were performed with a Bruker D8 ADVANCE diffractometer in Bragg-Brentano geometry with Cu Kα radiation. A GSAS program and EXPGUI interface were used for the Rietveld refinement (*51*). TEM was performed on JEOL 2100F with UHR configuration. The EELS were collected with Gatan 963 Quantum GIF SE, and the spectrum was processed with GMS3 software. The XPS tests were performed using PHI-5400 equipment with an Al Ka beam source (250 W) and a position-sensitive detector. An XPSpeak41 software is applied for peak fitting. The BET surface areas were measured with nitrogen adsorption-desorption tests (ASAP TriStar II 3020).

### XAS measurements and simulation

The samples for ex situ XAS measurements were collected by performing the tests on a large working electrode with increased catalyst loading. Specifically, 50 mg of catalyst was loaded onto a large carbon paper (3 cm by 3 cm). The cycling tests were performed in a three-electrode system (single cell) without electrode rotating. The hard XAS measurements at Ir L-edge and Co K-edge were performed at beamline 9-BM of the Advanced Photon Source (APS) at Argonne National Laboratory. The Athena and Artemis software packages were used for data analysis. Soft XAS measurements (O K-edge) were carried out at 4-ID-C at APS. Calculations of the O K-edge X-ray absorption near edge structure (XANES) were performed using the finite difference method as implemented within the finite difference method near-edge scattering (FDMNES) package using a free-form self-consistent field (SCF) potential of radius 6.0 Å around the absorbing atom (*52*). Broadening contributions due to the finite mean-free path of the photoelectron and to the core-hole lifetime were accounted for using an arctangent convolution.

### TOF calculation

The overpotential required to reach a TOF of 0.03 s^−1^ is obtained from Fig. 4B by calculating the corresponding current density of *j*_ECSA_ (normalized to ECSA). *j*_ECSA_ is estimated with the equation

$$j_{\text{ECSA}}=\text{TOF}\times 4\times e\times \rho_{\text{Ir}}$$

where *e* is the electric charge carried by a single electron. ρ_Ir_ is the surface density of Ir atoms. While calculating the ρ_Ir_, different surface atom arrangements are considered. For IrO_2_ (110) film with a stable surface, the (110) facet is considered and the lattice parameters are from Sen *et al*. (*53*). For SSI-OH with a stable surface, we assume that the B-site Sc and Ir are fully ordered to simplify the calculation. Two cases of the (100) facet (with the lowest Ir density) and (001) facet (with the highest Ir density) are considered. The refined lattice parameters from table S2 are used for calculations. For SCI-OH, SSI-H, and SCI-H with reconstructed surfaces, we consider two optimized structures of H_2_IrO_3_ (honeycomb) and IrOOH (brucite). In both structures, the (001) facet (with the highest Ir density) is used. The lattice parameters are obtained from DFT calculations.

### DFT calculations

The spin-polarized DFT calculations were performed using the Vienna Ab initio Simulation Package (*54*), using the projected augmented wave model. The exchange and correlation effect was described by the Perdew-Burke-Ernzerhof functional (*55*). The generalized gradient approximation (GGA) + *U* calculations were performed using the model proposed by Dudarev *et al.* (*56*), with *U*_eff_ (*U*_eff_ = Coulomb *U* − exchange *J*) values of 1, 3.3, and 3 eV for Ir, Co, and Sc, respectively (*53*, *57*, *58*). In all the calculations, the cutoff energy was set to 450 eV. The Monkhorst-Pack (*59*) *k*-point meshes were set to 6 × 6 × 5 and 2 × 2 × 1 for performing the bulk and surface calculations of the perovskite structure, respectively. The force and energy convergence tolerance were set to 0.05 eV Å^−1^ and 10^−5^ eV, respectively.

The OER free energies were calculated on the basis of the following four elementary steps

$$\begin{array}{c} {\text{OH}}^{–}+*\to *\text{OH}+e^{-}\\ {}*\text{OH}+{\text{OH}}^{-}\to *O+H_{2}O+e^{-}\\ {}*O+{\text{OH}}^{-}\to *\text{OOH}+e^{-}\\ {}*\text{OOH}+{\text{OH}}^{-}\to *+O_{2}+H_{2}O+e^{-} \end{array}$$

where * denotes the cation sites on the catalyst surface. On the basis of the above mechanism, the free energies of the three intermediate states—*OH, *O, and *OOH—are crucial in determining the OER activity of a given material. The computational hydrogen electrode (CHE) model (*60*) was used to evaluate the energy state of the OER intermediates, based on which the free energy of an adsorbed species is defined as

$$∆G_{\text{ads}}=∆E_{\text{ads}}+∆E_{\text{ZPE}}-T∆S_{\text{ads}}$$

where ∆*E*_ads_ is the electronic adsorption energy, ∆*E*_ZPE_ is the zero-point energy difference between adsorbed and gaseous species, and *T*∆*S*_ads_ is the corresponding entropy difference between these two states. The electronic binding energy is referenced as ^1^/_2_ H_2_ for each H atom, and (H_2_O-H_2_) for each O atom, plus the energy of the clean slab. The corrections of zero-point energy and entropy of the OER intermediates can be found in table S6.

The surface Pourbaix diagram was calculated on the basis of the method proposed by Hansen *et al.* (*61*), where the free energy of oxygen and hydroxyl exchange at a given surface at any pH and potential is calculated as

$$G(\text{HO}*)=\Delta G_{0}(\text{HO}*)-eU_{\text{SHE}}-k_{B}T\text{ln}10\text{pH}+\Delta G_{\text{field}}$$

where Δ*G*_field_ is the change in the adsorption energy due to the electric field in the electrochemical double layer at the cathode. According to the work by Rossmeisl *et al.* (*62*), the relative stability change in O* and OH* under an electric field is more than one order magnitude lower than the change in free energy. Therefore, it is believed that the trend in adsorption energies can be well described by neglecting Δ*G*_field_ in the construction of the surface Pourbaix diagram.

## Supplementary Materials

### This PDF file includes:

Figures S1 to S23 Tables S1 to S6 References
