# Supplementary material for: Lattice site–dependent metal leaching in perovskites toward a honeycomb-like water oxidation catalyst
Source: Sci Adv. 2021 Dec 10;7(50):eabk1788. doi: 10.1126/sciadv.abk1788 (PMC8664262; doi:10.1126/sciadv.abk1788)
Supplement: Supplementary file 1 — Figures S1 to S23 Tables S1 to S6 References [file sciadv.abk1788_sm.pdf]

Science Advances
AAAS

Supplementary Materials for

Lattice site–dependent metal leaching in perovskites toward a honeycomb-like water oxidation catalyst

Yubo Chen, Yuanmiao Sun, Maoyu Wang, Jingxian Wang, Haiyan Li, Shibo Xi, Chao Wei, Pinxian Xi, George E. Sterbinsky, John W. Freeland, Adrian C. Fisher, Joel W. Ager III, Zhenxing Feng*, Zhichuan J. Xu*

*Corresponding author. Email: zhenxing.feng@oregonstate.edu (Z.F.); xuzc@ntu.edu.sg (Z.J.X.)

Published 10 December 2021, *Sci. Adv.* **7**, eabk1788 (2021)
DOI: 10.1126/sciadv.abk1788

This PDF file includes:

Figures S1 to S23
Tables S1 to S6
References

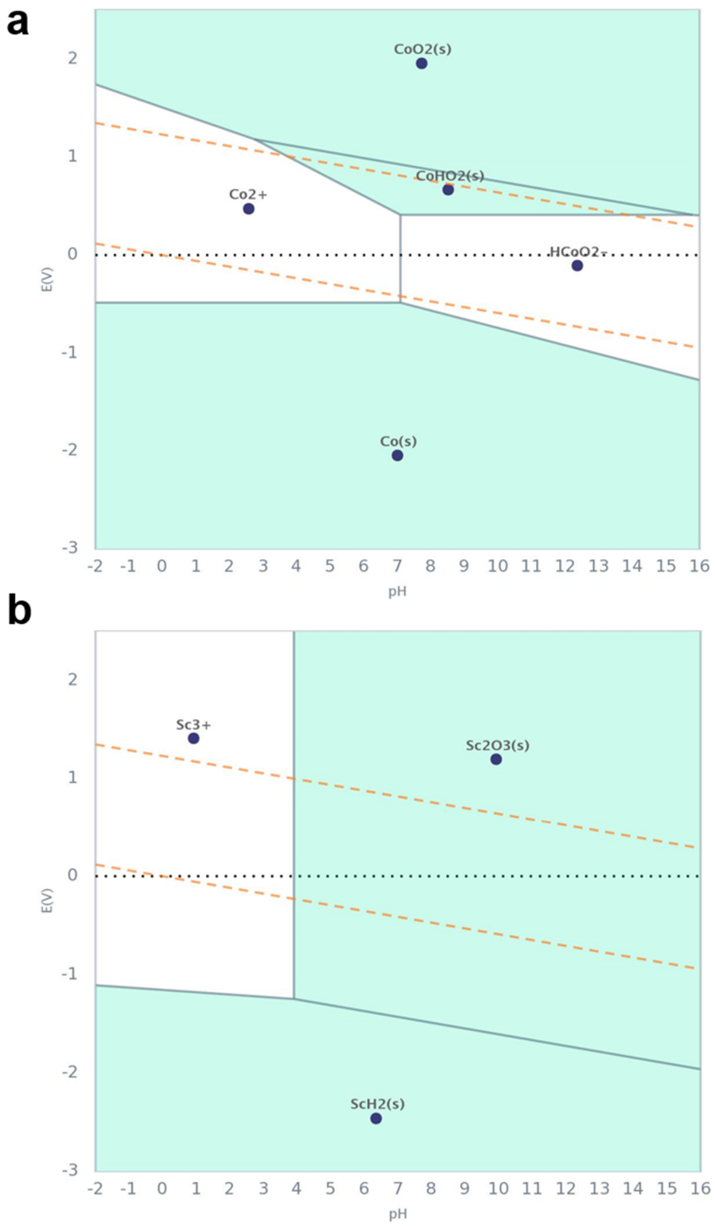

Fig. S1.

Pourbaix Diagrams for cobalt and scandium | The data are from the *materialsproject.org* and the concentration of ions is 10^{-6} M. (*63*, *64*) For Co (**a**), it is thermodynamically unstable at both high pH ($HCoO^{2-}$) values and low pH (Co^{2+}) values. For Sc (**b**), it is thermodynamically stable at high pH (Sc_2O_3) values but unstable at low pH (Sc^{3+}) values.

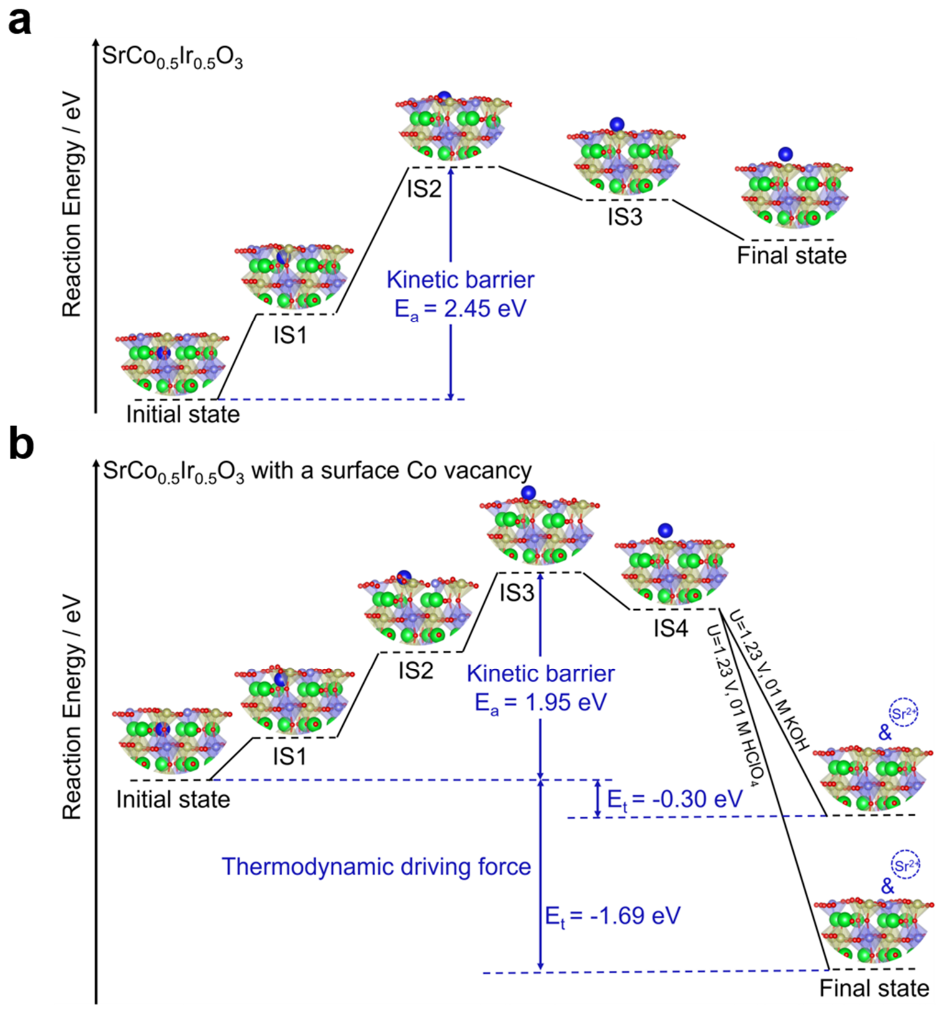

Fig. S2.

Theoretical prediction of $SrCo_{0.5}Ir_{0.5}O_3$ surface stability | The energy diagrams that illustrate the dissolution of A-site (Sr) from the sub-surface of $SrCo_{0.5}Ir_{0.5}O_3$ without (**a**) and with (**b**) a B-site (Co) vacancy. We note that the dissolution of outer surface Sr into the electrolyte is not considered for $SrCo_{0.5}Ir_{0.5}O_3$ surface without Co vacancy. This is because that Co cannot be stable in either high or low pH values.

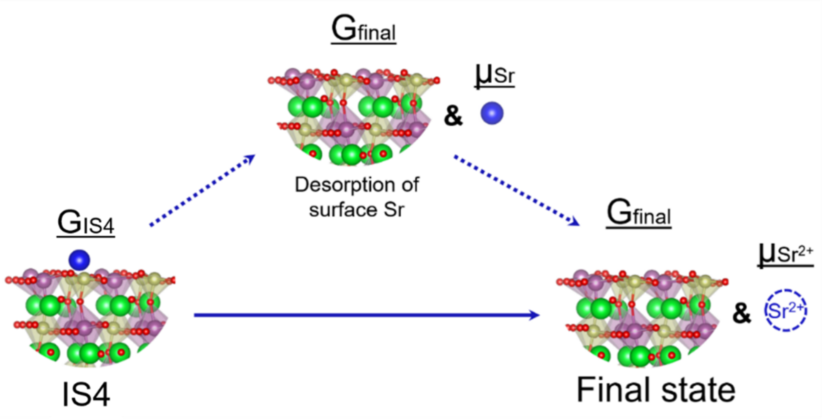

Fig. S3.

Dissolution of outer-surface Sr | The schematic for the dissolution of lattice Sr, from the outer-surface, into the electrolyte as Sr^{2+} ion.

As shown in **Fig. S3**, the second stage of A-site Sr dissolution (IS4 to final state) includes two sub-steps. In the first sub-step, A lattice Sr, which migrates from the sub-surface to the outer surface, leaves the surface as a single atomic Sr. The free-energy change for this step (ΔG_1) can be expressed as

$$\Delta G_1 = G_{final} + \mu_{Sr} - G_{IS4} \quad (1)$$

The G_{IS4} and G_{final} are the free energies of the surfaces before and after the desorption of surface Sr, respectively. μ_{Sr} is the chemical potential of the Sr atom. Such chemical potential is estimated by calculating the chemical potential of a Sr metal model, in which a face-centered cubic Sr crystal is constructed for calculation. The second sub-step is the dissolution of atomic Sr into the electrolyte ($Sr = Sr^{2+} + 2e^-$). The free-energy change for the dissolution (ΔG_2) can be expressed as

$$\Delta G_2 = \mu_{Sr^{2+}} + 2\mu_{e^-} - \mu_{Sr} \quad (2)$$

$\mu_{Sr^{2+}}$ and μ_{e^-} are the chemical potential of Sr^{2+} and e^-. The ΔG_1 for the first step is calculated based on DFT. The calculation details are discussed in the method. The ΔG_2 is calculated with the standard hydrogen electrode as the reference.(*29*) Under the operational condition, the chemical potential of $\mu_{Sr^{2+}}$ and μ_{e^-} can be correlated to the standard states by

$$\mu_{Sr^{2+}} = \mu^{\circ}_{Sr^{2+}} + kTlna_{Sr^{2+}} \quad (3)$$

$$\mu_{e^-} = \mu^{\circ}_{e^-} - eU_{SHE} \quad (4)$$

Substitution of the above two equations into equation (2) gives

$$\Delta G_2 = \Delta G^{\circ}_{SHE} - 2eU_{SHE} + kTlna_{Sr^{2+}} \quad (5)$$

, where ΔG°_{SHE} and $a_{Sr^{2+}}$ are standard hydrogen electrode free energy of Sr and the Sr^{2+} ion concentration. For Sr, the ΔG°_{SHE} value is -5.8 eV and the $a_{Sr^{2+}}$ is fixed at 10^{-6} M.(*65*)

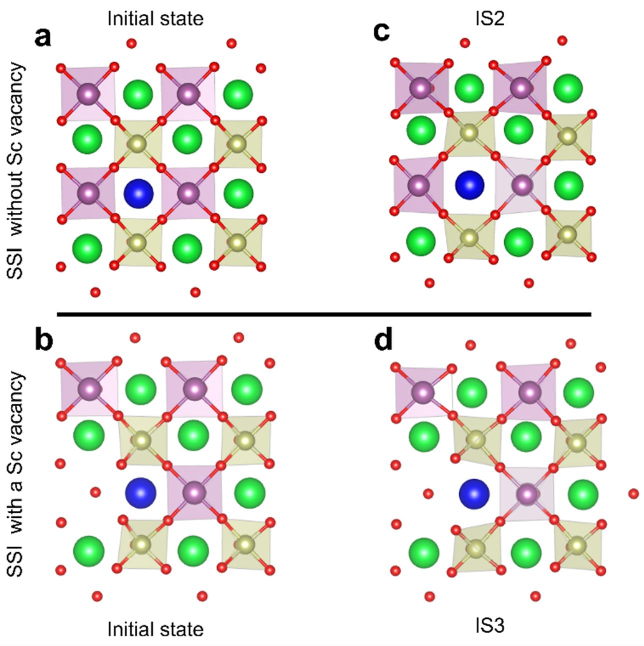

Fig. S4.

Migration of lattice Sr from sub-surface to outer-surface | The top view of the initial (**a&b**) and intermediate states (**c&d**) of model SSI with and without a Sc vacancy. For a better expression, only two atom layers of surface B-site layer and sub-surface A-site layer are shown. The intermediate state 2 (IS2) and intermediate state 3 (IS3) are selected for SSI and SSI with an Sc vacancy, respectively.

The Sr will move through an aperture constructed by four (**Fig. S4c** without Sc vacancy) or three (**Fig. S4d** with an Sc vacancy) surface corner-shared BO5 square pyramids. Due to the large ionic size of Sr, the surface atoms must relax substantially to allow the migration of Sr, hinting at the existence of steric hindrance for Sr migration. This is reflected in the distorted BO5 square pyramids in IS2 from SSI and IS3 from SSI with an Sc vacancy. Due to the existence of an Sc vacancy, the BO5 square pyramids in **Fig. S4d** can distort more freely to make more space available for Sr migration. As a result, a lower kinetic barrier for Sr migration is found from the SSI with an Sc vacancy.

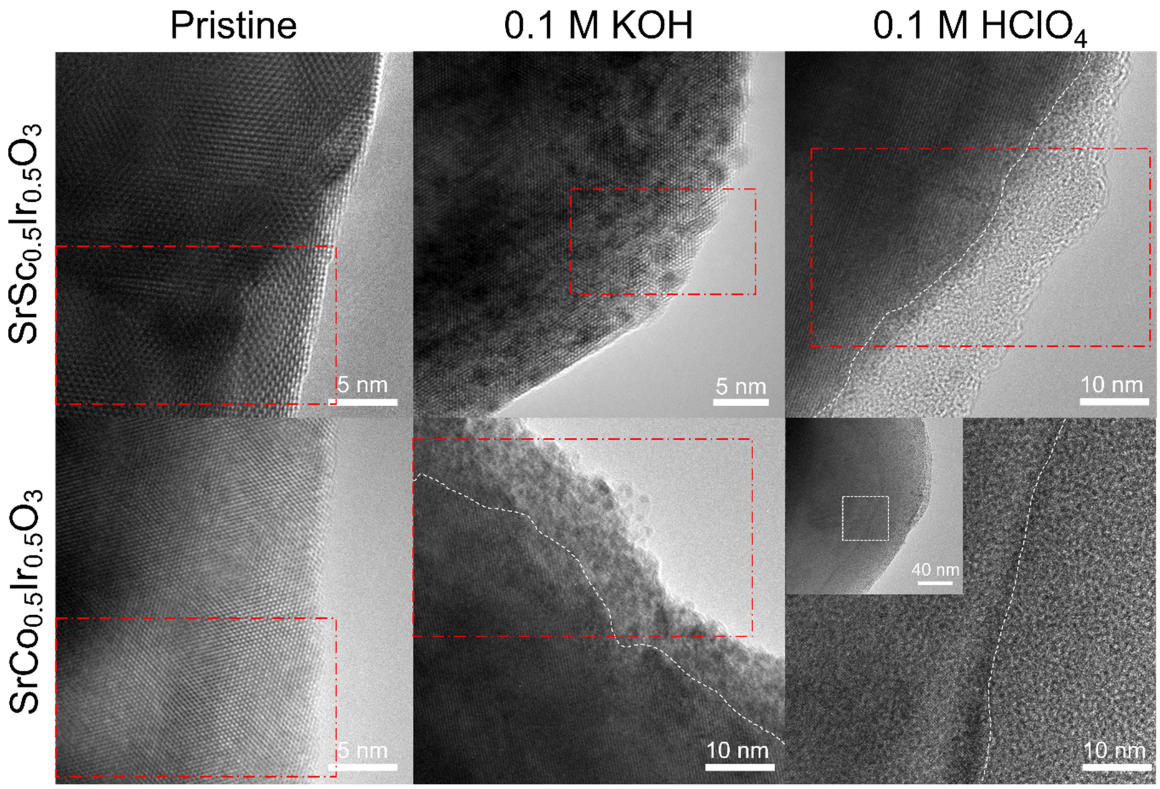

Fig. S5.

Crystal structure of perovskite surfaces | HR-TEM images of the surface crystal structure of two model perovskites before (pristine) and after cycling in 0.1 M KOH and 0.1 M $HClO_4$. The regions highlighted in red are enlarged and presented in the main text.

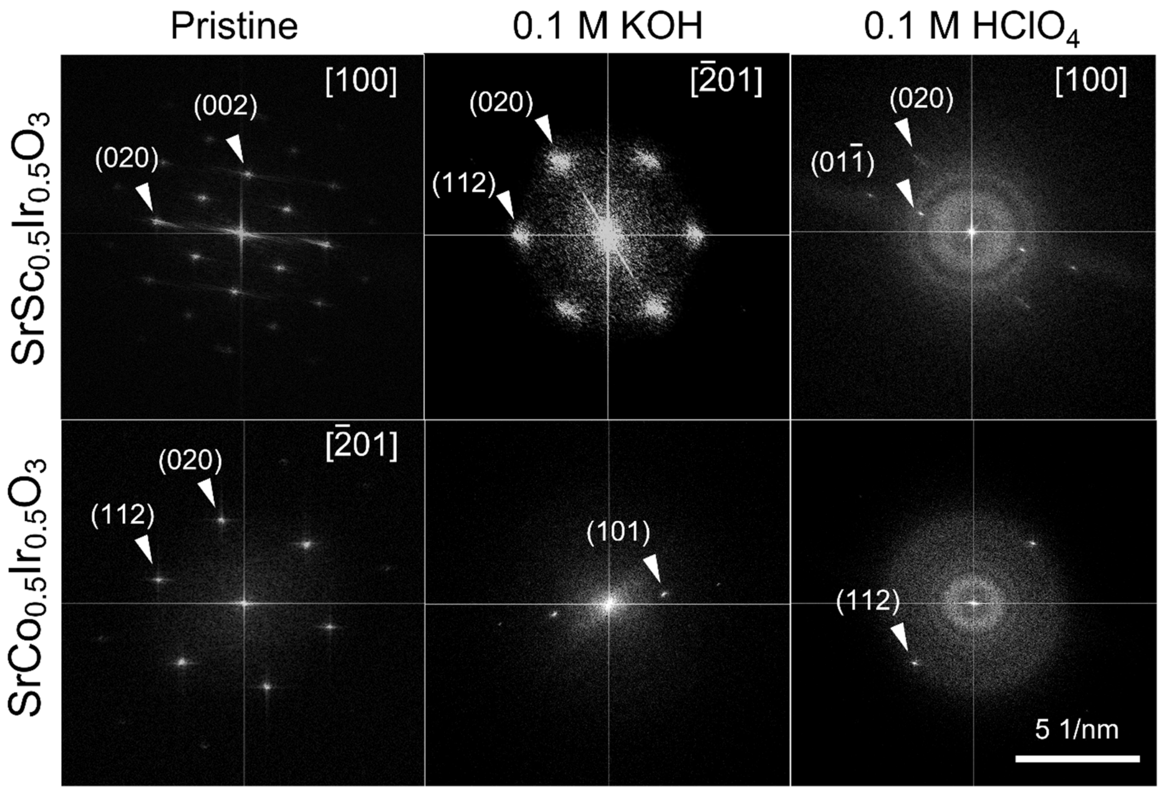

Fig. S6.

Index of perovskite surface structures | The corresponding FFT images of the TEM images in **Fig. S5**.

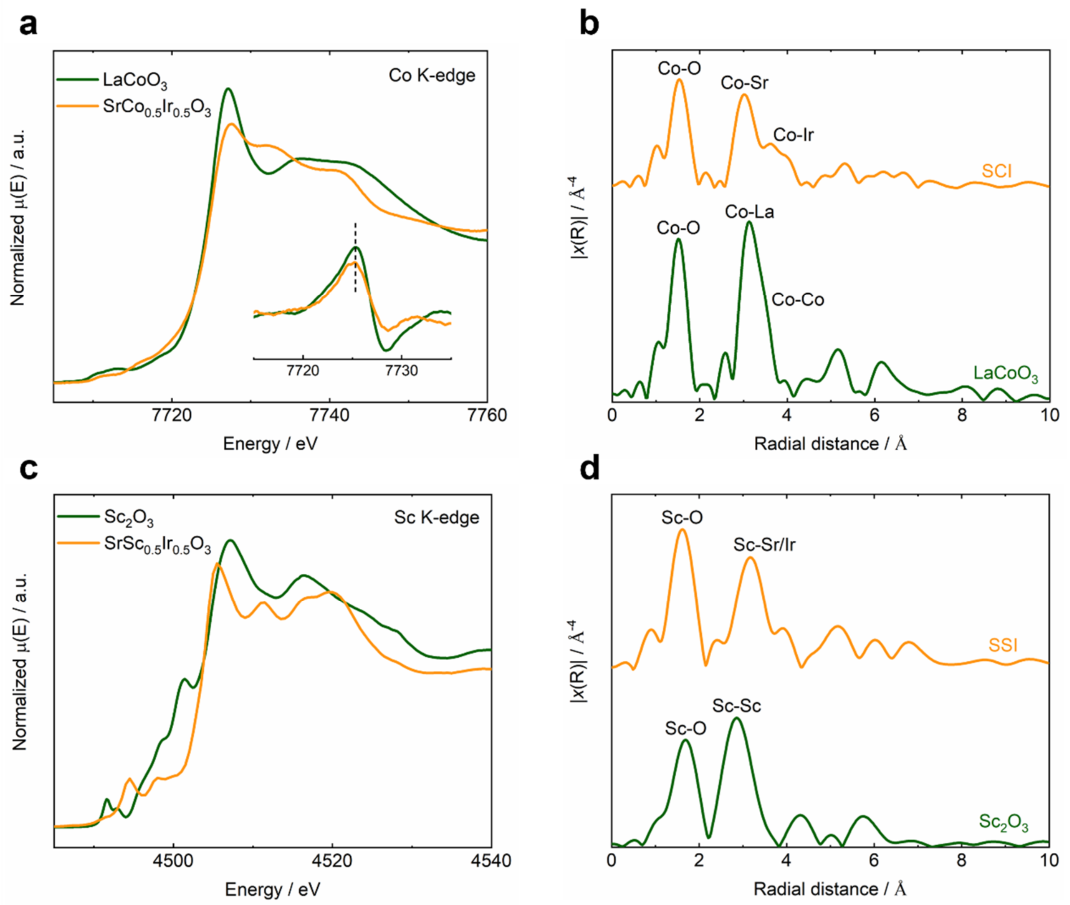

Fig. S7.

Status of Co and Sc in perovskite structure | The Co K-edge XANES spectra (**a**) and EXAFS spectra (**b**) from $LaCoO_3$ and $SrCo_{0.5}Ir_{0.5}O_3$. The inset in (**a**) is the corresponding first derivative. The Sc K-edge XANES spectra (**c**) and EXAFS spectra (**d**) from Sc_2O_3 and $SrSc_{0.5}Ir_{0.5}O_3$.

As shown in **Fig. S7a**, the positions of the white line from $LaCoO_3$ and SCI are close to each other, confirming that the trivalent Co is dominant in SCI. The Co is demonstrated in SCI perovskite structure as the featured peaks of Co-Sr and Co-Ir, related to SCI perovskite structure, can be identified in **Fig. S7b**. From the normalized Sc K-edge XANES spectra (**Fig. S7c**), the profile of Sc K-edge XANES spectra from SSI is much different from the case in Sc_2O_3, hinting that the Sc can be in a perovskite structure. This is because that the profile of Sc K-edge XANES spectra is found highly sensitive to the local structural environment.(*66*) The Sc from SSI in perovskite structure is further demonstrated by the corresponding EXAFS spectra in **Fig. S7d**. The peak of Sc-Sr, related to perovskite structure, can be observed.

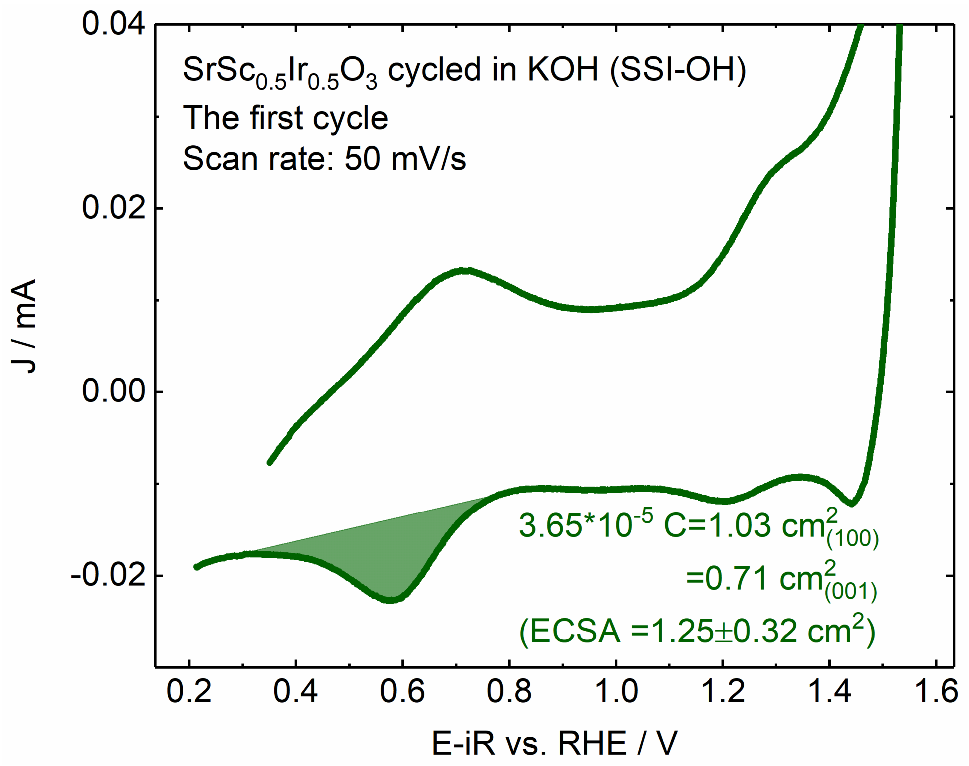

Fig. S8.

Estimation of the electrochemical surface area of SSI-OH | The first CV cycle of $SrSc_{0.5}Ir_{0.5}O_3$ in alkaline condition (SSI-OH). The conversion of redox charge to surface area is realized with the Ir atom density in crystalized perovskite surface with different exposed facets. The surface Ir density (ρ_{Ir}) is $2.217 \times 10^{14}\ Ir/cm^2$ and $3.225 \times 10^{14}\ Ir/cm^2$ for the (100) facet and (001) facet, respectively. The corresponding surface area (A) can be calculated with

$$A = \frac{Q}{e \times \rho_{Ir}}$$

Where Q is the integral charge from redox peak (Ir^{4+} to Ir^{3+}), e is the charge of a single electron. The calculated surface area is 1.03 cm^2 and 0.71 cm^2 for the (100) facet and (001) facet, respectively. These two surface areas are close to the measured ECSA of 1.25±0.32 cm^2 from impedance analysis (**Fig. S12**), indicating that only the Ir from the outer perovskite surface is involved.

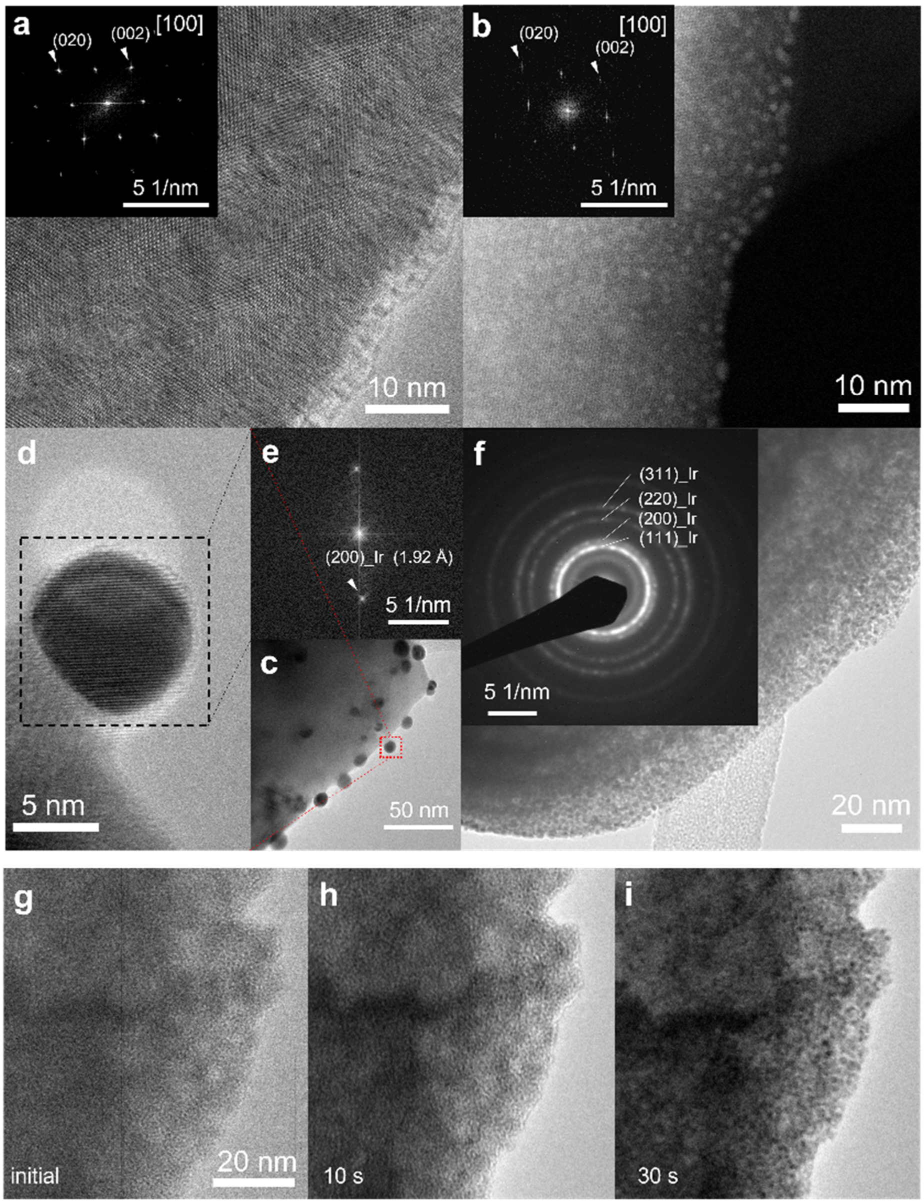

Fig. S9.

The evolution of Ir nanoparticles from perovskite surfaces | The HR-TEM (**a**) and STEM (**b**) image of the pristine $SrCo_{0.5}Ir_{0.5}O_3$ with electron beam illumination. Insets are the corresponding FFT images. (**c**) The TEM image of the $SrCo_{0.5}Ir_{0.5}O_3$ surface with the formation of nanoparticles. (**d**) The local structure of the nanoparticle. The corresponding FFT image (**e**) can be indexed with Ir metal. (**f**) The TEM image of the $SrCo_{0.5}Ir_{0.5}O_3$ cycled in acid. After electron beam illumination, many nanoparticles evolved from the reconstructed amorphous surface. The inset is the SAED pattern from the surface region. This SAED pattern can also be indexed with Ir metal, confirming the electron beam illumination induces the formation of Ir nanoparticles. (**g-i**) The evolution of TEM images taken from the reconstructed surface of $SrCo_{0.5}Ir_{0.5}O_3$ cycled in acid. Images were taken initially, 10 s and 30 s. It can be found that, after approximately 30s of electron beam exposure, numerous black Ir nanoparticles evolve.

Note that apart from electron beam induced formation of Ir nanoparticles, the reconstructed perovskite surfaces from SCI-OH, SSI-H, and SCI-H in this work are strictly amorphous. Nevertheless, the strict amorphization or formation of Ir/IrO_2 nanoparticles at the surface of Ir-based perovskite oxides is under debate.(*13, 16, 25, 33*) For example, the formation of rutile IrO_2 nanoparticles have been reported from the reconstructed surfaces of a pseudocubic $SrIrO_3$ (with corner-shared IrO6 octahedrons) thin films. It is found that IrO_2-related nanocrystallites form in five anodic cycles but disappear when the number of anodic cycles increases to 130.(*16*) However, with similar electrochemical tests, the reconstructed surface of a polycrystalline monoclinic $SrIrO_3$ (with mixed edge-/corner-shared octahedrons) is strictly amorphous and no nanocrystallites can be observed.(13, 16) Given their identical composition, the much difference in crystal structure may account for the formation of reconstructed surfaces with different properties. This deduction is also supported by another work, in which the reconstructed Ir-based surface is found to be more active if the IrO6 octahedrons are corner-shared in the initial perovskite structure.(*13*) In this study, the IrO6 octahedrons in the initial SCI and SSI are also corner-shared. However, the IrO_2-related nanocrystallites cannot be found over the reconstructed surfaces. It is possible that the presence of foreign B-site metals (Co and Sc) prohibits the evolution of rutile IrO_2.

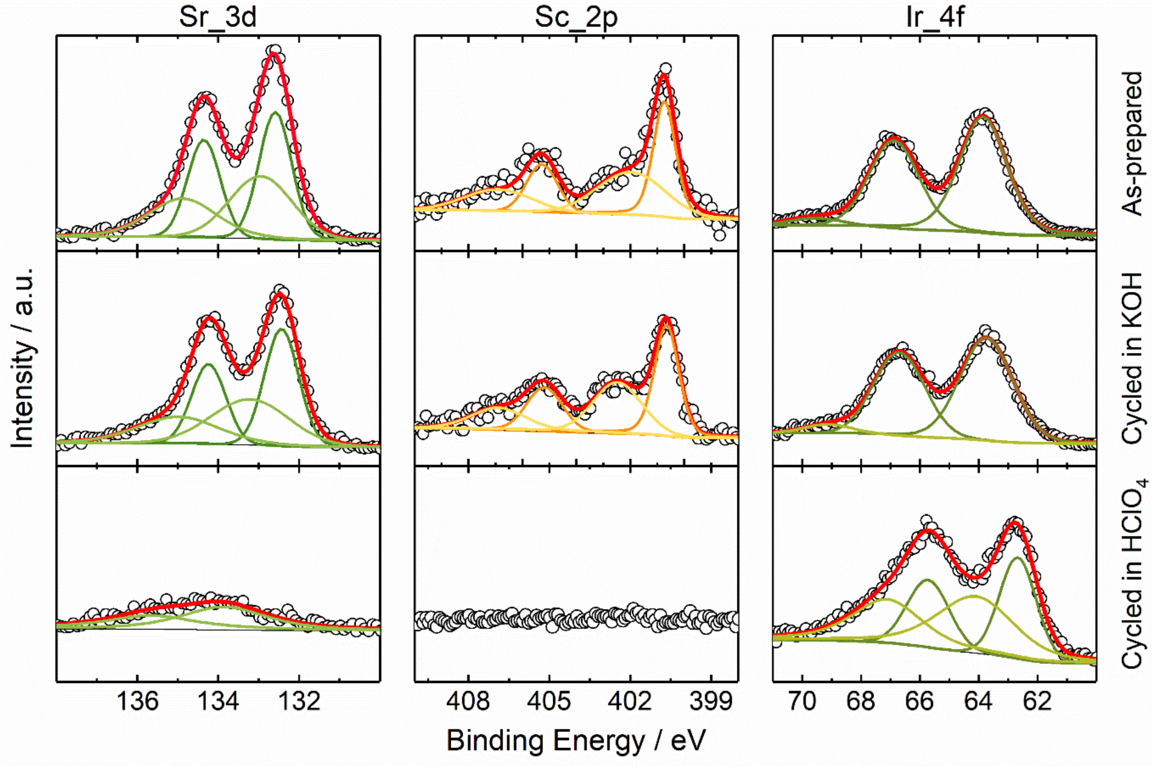

Fig. S10.

Variation of SSI surface composition | The XPS spectra from as-prepared SSI, SSI cycled in KOH, and SSI cycled in $HClO_4$. The fitting parameters are listed in **Table S4.**

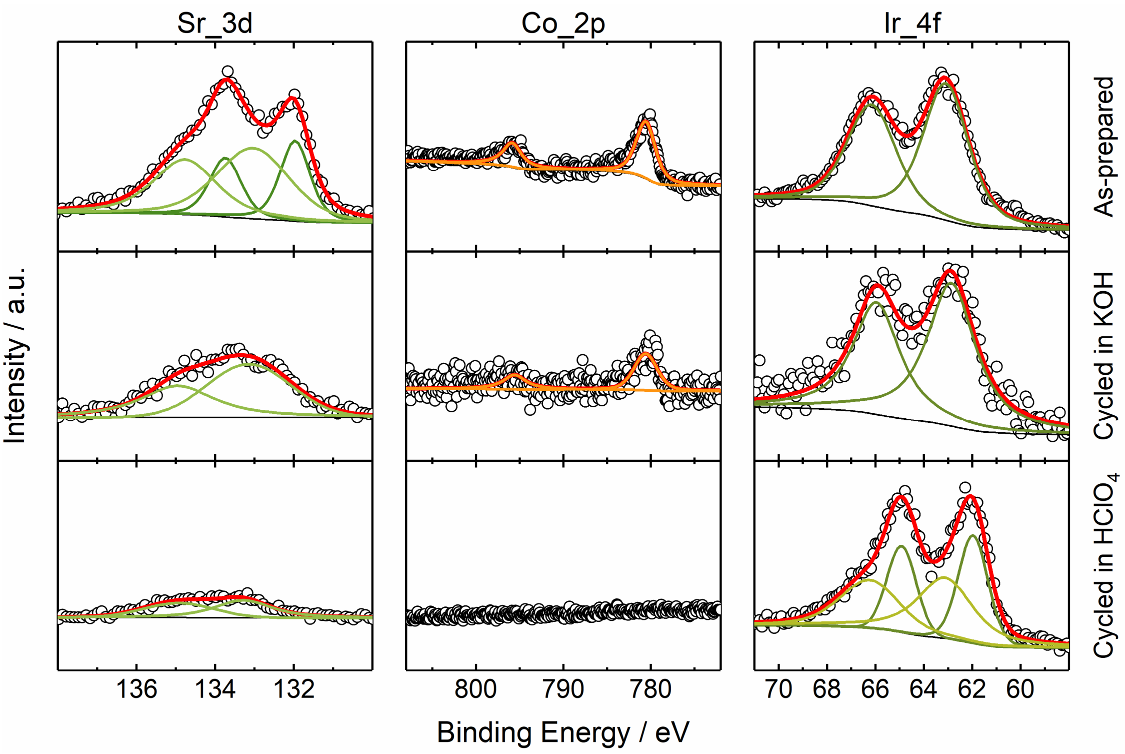

Fig. S11.

Variation of SCI surface composition | The XPS spectra from as-prepared SCI, SCI cycled in KOH, and SCI cycled in $HClO_4$. The fitting parameters are listed in **Table S5.**

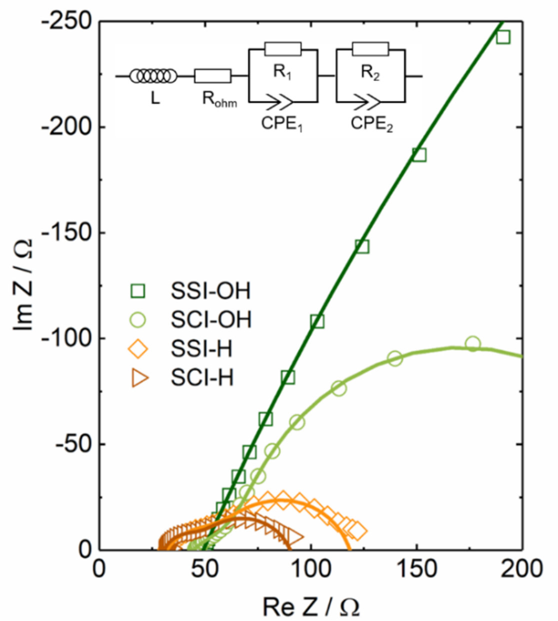

Fig. S12.

Calculation of ECSA with EIS | Three reprehensive impedance spectra from SSI-OH, SCI-OH, SSI-H, and SCI-H. The tests are performed at a potential of 1.5 V vs. RHE. Specifically, during the CV tests, the corresponding electrochemical impedance measurements are conducted at the beginning (after 2 CV cycles) for SSI-OH and after 50 CV cycles for SCI-OH, SSI-H, and SCI-H. This is because that the OER current in the initial CV cycles of SSI-OH is originated from the near-ideal SSI perovskite surface and the OER currents in the final CV cycles of the other three samples are originated from the fully reconstructed surface. Inset is the equivalent circuit ($LR_{ohm}(R_1//CPE_1)(R_2//CPE_2)$) for identifying the charge transfer process and diffusion process. The L and R_{ohm} represent the inductance and ohmic resistance of the testing system, respectively. The parallel $R_1//CPE_1$ and $R_2//CPE_2$ correspond to the charge transfer process and diffusion process, respectively. Specifically, in the charge transfer process, the R_1 is the charge transfer resistance and the CPE_1, a constant phase element, is used in place of a capacitor to compensate for non-homogeneity in the system.

The CPE can be expressed as

$$Z_{CPE} = \frac{1}{T(Iw)^\wedge P}$$

Where T is frequency-independent constant with the unit of $F\ s^{(P-1)}m^{-2}$, I is the square root (-1), w is the angular frequency of the AC signal, and P is a parameter ranging from 0 to 1. The electrochemical double-layer capacitance can be obtained with the equation of(*41*)

$$C = (T(\frac{1}{R_{ohm}} + \frac{1}{R_1 + R_2})^{P-1})^{1/P}$$

The electrochemical surface area can be calculated with

$$ECSA = \frac{C}{C_s}$$

The C_s is the specific capacitance of the sample. The selection of specific capacitance is discussed in "**Additional discussion of C_s selection**".

Additional discussion of C_s selection

In **Fig. 4b** of the main text, a typical specific capacitance of 0.4 $\mathrm{F\ m^{-2}}$ is used for estimating the ECSA for SSI-OH, SCI-OH, SSI-H, and SCI-H. This value is an average specific capacitance, which is derived from the reported specific capacitances of various metallic surfaces.(*2*) The estimated ECSA based on this typical specific capacitance for activity normalization can be fairly reliable. This specific capacitance is demonstrated proper for SSI-OH with a stable surface. This is because that the estimated ECSA is close to the area estimated with redox charge (**Fig. S8**). For SCI-OH, SSI-H, and SCI-H with reconstructed amorphous surfaces, a similar specific capacitance is expected. For instance, with the standard specific capacitance of 0.4 $\mathrm{F\ m^{-2}}$, the normalized OER current densities of SSI-H and SCI-H are close to each other (**Fig. 4b**). This corresponds well with the conclusion that a similar phase is formed over their reconstructed surfaces (**Fig. S19**). Nevertheless, for SCI-OH, SSI-H, and SCI-H with reconstructed surfaces, using such a specific capacitance of 0.4 $\mathrm{F\ m^{-2}}$ may induce under- or over-estimated intrinsic activity.

Based on the literature reports, the specific capacitance of metal electrodes can change from 0.15 $\mathrm{F\ m^{-2}}$ to 1.1 $\mathrm{F\ m^{-2}}$ in H_2SO_4 and from 0.22 $\mathrm{F\ m^{-2}}$ to 1.3 $\mathrm{F\ m^{-2}}$ in NaOH and KOH electrolytes.(*2*) For rutile IrO_2, a specific capacitance of 1.3 $\mathrm{F\ m^{-2}}$ is also reported.(*41*) Thus, we also check the ECSA-normalized OER current densities with the consideration of specific capacitance variation (**Fig. S13**). For SCI-OH, specific capacitances from 0.22 $\mathrm{F\ m^{-2}}$ to 1.3 $\mathrm{F\ m^{-2}}$ are considered. For SSI-H and SCI-H, specific capacitances from 0.15 $\mathrm{F\ m^{-2}}$ to 1.1 $\mathrm{F\ m^{-2}}$ are considered.

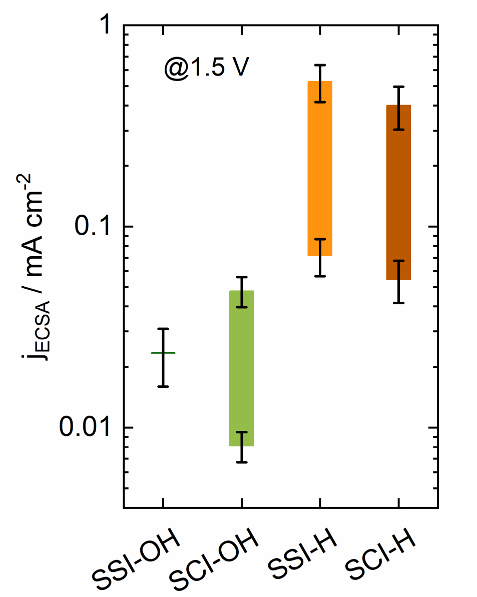

Fig. S13.

OER currents with the consideration of C_s variation | The OER current (normalized to ECSA) densities at 1.5 V from all four samples. The ECSA is calculated with the consideration of C_s variation.

Irrespective of large error induced by specific capacitance, the intrinsic current densities of SSI-H and SCI-H are substantially higher than the intrinsic current densities of SSI-OH and SCI-OH, confirming the promoting effect of surface reconstruction and the importance of B-site metal leaching.

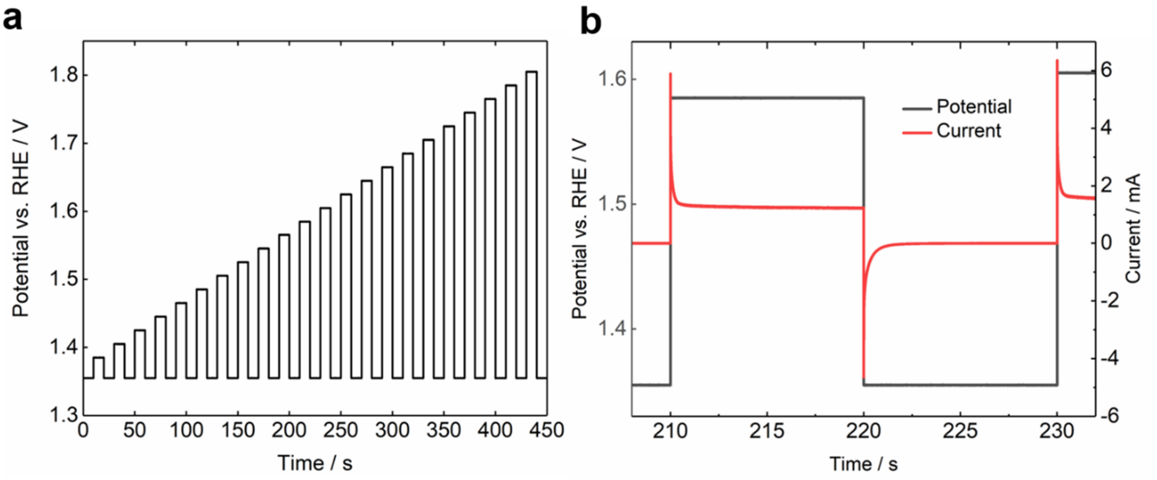

Fig. S14.

Details of pulse voltammetry protocol | (a), The potential step applied in pulse voltammetry protocol. The potential vs. RHE changes between 1.35 V (cathodic) and 1.42 V to 1.8 V (anodic). The potential holds for 10 s for each step. (**b)**, The current response of a typical anodic and cathodic section. The OER current is from the SCI-H.

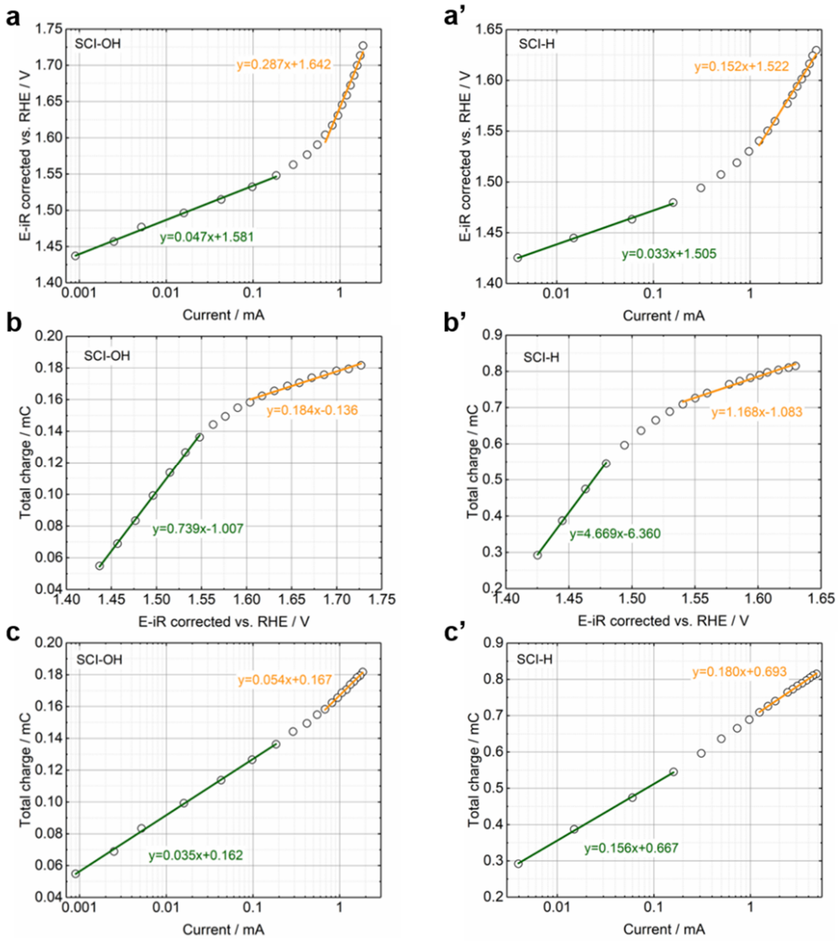

Fig. S15.

Correlations between current response and total charge | The measured current response and total charge of SCI-OH (**a-c**) and SCI-H (**a'-c'**) from pulse voltammetry. (**a&a')**, The Tafel plots of potential (iR corrected vs. RHE) vs. logarithm of OER current. For SCI-OH with A-site cation leaching, the bending starts at a potential of ~1.55 V. For SCI-H with additional B-site cation leaching, the bending starts at a potential of ~1.48 V. (**b&b')**, The total charge (integral cathodic charge) vs. potential (iR corrected vs. RHE). (**c&c'),** The total charge vs. logarithm of OER current.

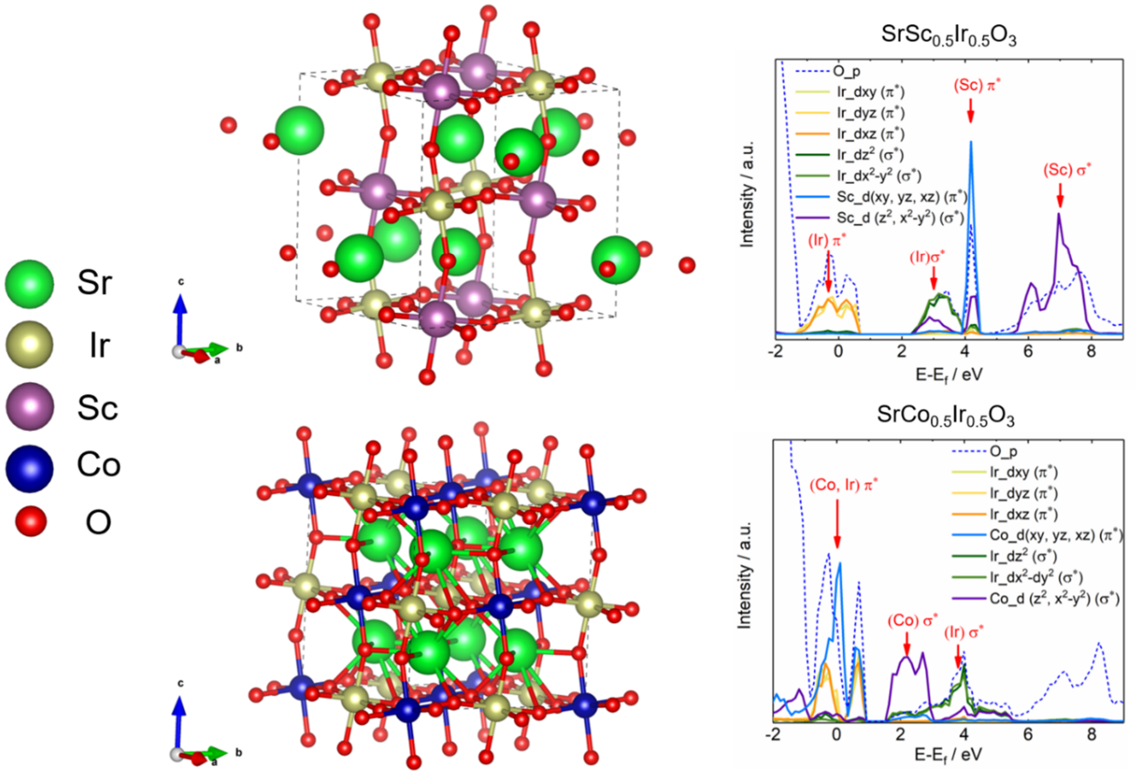

Fig. S16.

Calculated electronic structures of model perovskites | The crystal structure of $SrSc_{0.5}Ir_{0.5}O_3$ and $SrCo_{0.5}Ir_{0.5}O_3$ and the corresponding PDOS of Ir_d and O_p states.

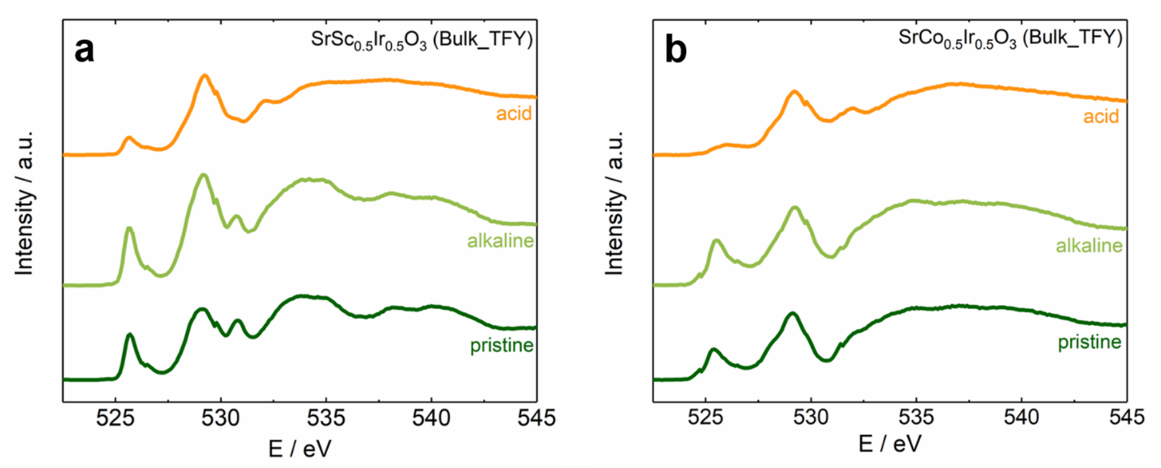

Fig. S17.

Measured O K-edge spectra from perovskite bulk | The O K-edge spectra from the pristine and electrochemically cycled $SrSc_{0.5}Ir_{0.5}O_3$ (**a**) and $SrCo_{0.5}Ir_{0.5}O_3$ (**b**). These XAS tests are performed in the total fluorescence yield model (TFY), which is more bulk sensitive compared with the XAS tests in the TEY model (**Figure 5**). The spectra from both samples, cycled in acid, are different from the ones from pristine samples. This corresponds well with the TEM results (**Fig. S5**) that the perovskite surface regions thoroughly reconstructed with increased depth in acid.

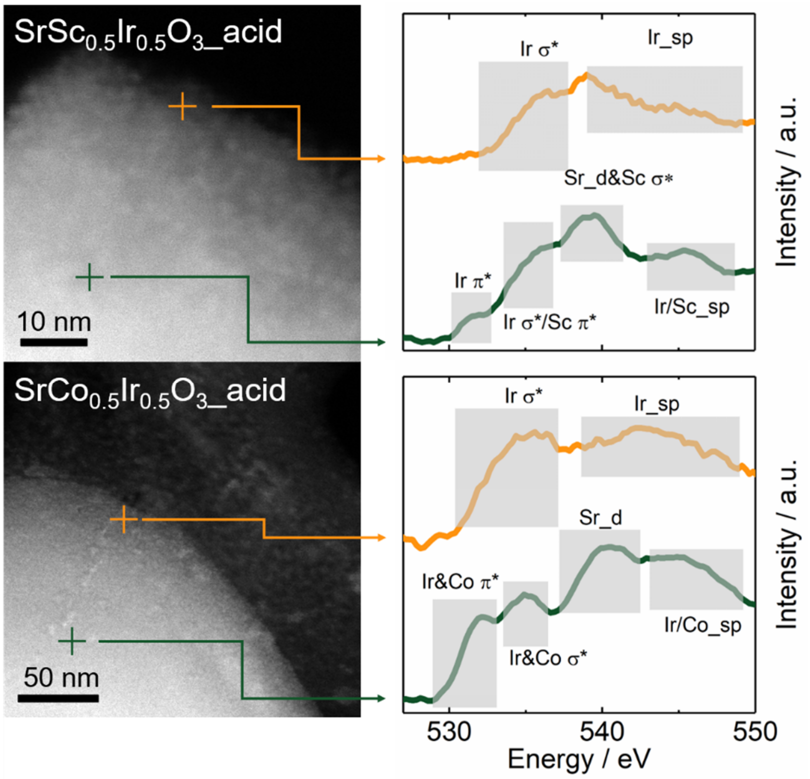

Fig. S18.

Measured O K-edge spectra from perovskite bulk and reconstructed surface | The O K-edge spectra of $SrSc_{0.5}Ir_{0.5}O_3$ and $SrCo_{0.5}Ir_{0.5}O_3$ from STEM-EELS analysis. Both samples are electrochemically cycled in 01 M $HClO_4$, and the signals are collected from either crystalized bulk or reconstructed outer surface. After surface reconstruction, the featured pre-edge peaks, related to π^*, cannot be observed. This should be related to its greatly reduced intensity (**Figure 5e-f**), which makes the collection of corresponding EELS signals difficult.

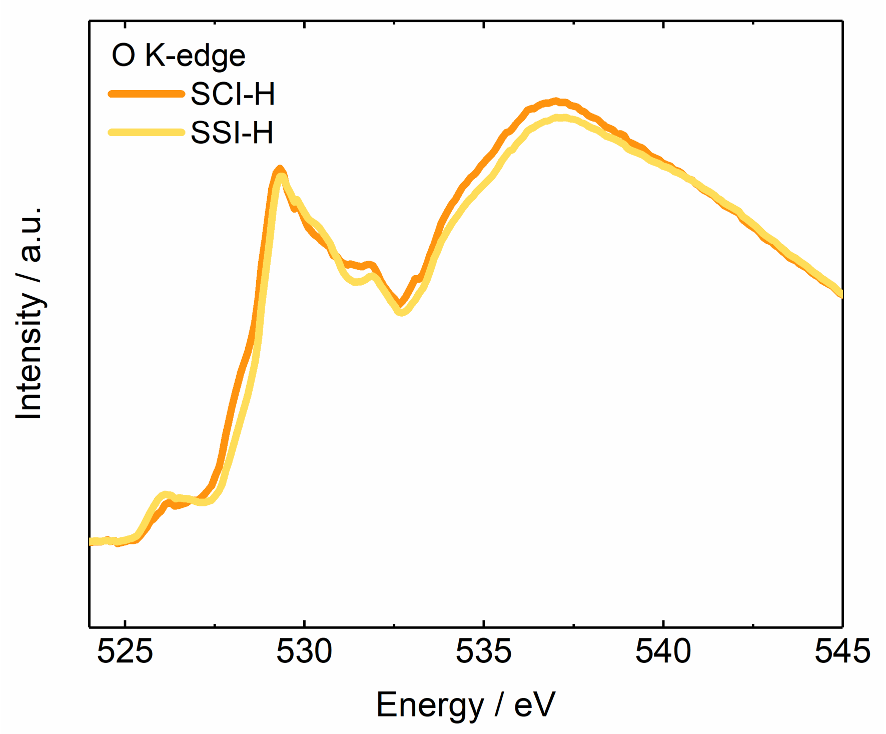

Fig. S19.

Comparison of O K-edge spectra | The O K-edge spectra from the reconstructed surfaces of SSI-H and SCI-H.

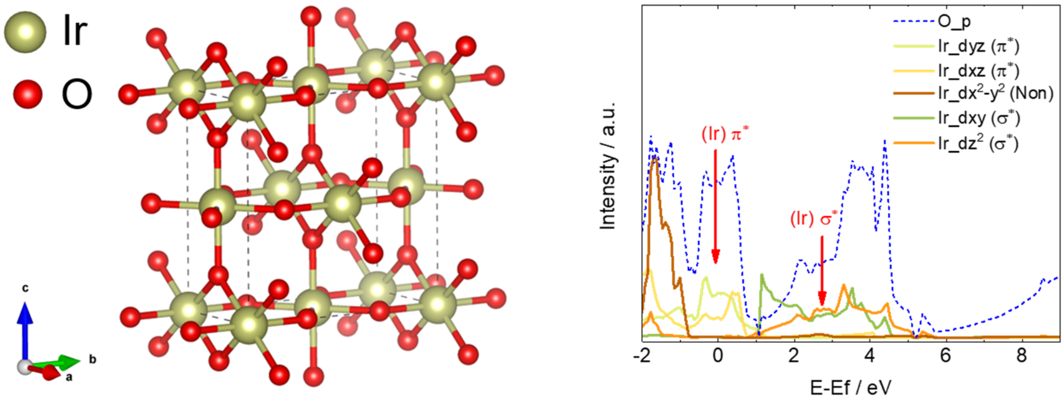

Fig. S20.

Calculated electronic structures of rutile IrO_2 | The crystal structure of IrO_2 and the corresponding PDOS of Ir_d and O_p states. The calculated PDOS is close to the one reported by Tobias Reier et al.(*24*)

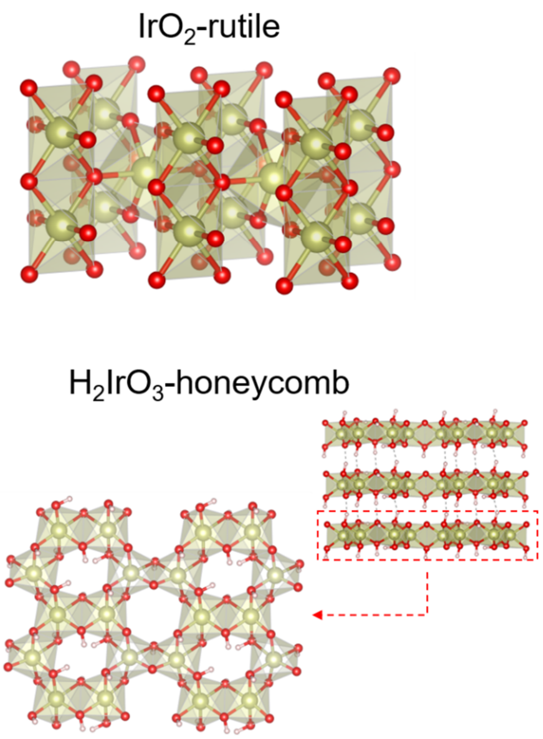

Fig. S21.

Possible structural motifs | A series of possible structures for the IrO_xH_y phase from the reconstructed perovskite surface. Based on the XAS results, four possible structures of IrO_2-rutile, H_2IrO_3-honeycomb, H_2IrO_3-F, and IrOOH-brucite are considered. Because the oxidation state of Ir in IrO_xH_y is close to 4 and the material should be electric neutrality, the value of *x* and *y* should fulfill the equation of $4 + y = 2x$. If $x = 2$, the stoichiometry is IrO_2. In this case, the IrO_2 with a rutile structure is possible. If $x \geq 3$, the proton should exist in the bulk. That is the formation of Ir-based (oxy)hydroxide. However, till now, only a layered IrOOH, with Ir^{3+}, has been reported.(*67*) Considering that Lithium should be the element most close to a proton, we turn to find more possible structures from the Li-Ir-O system. α-Li_2IrO_3 (space group: C *12/m1*) and β-Li_2IrO_3 (space group: F *ddd*) are found to be constructed with edge-shared IrO6 octahedrons. Thus, replacing Li with H, we proposed H_2IrO_3-honeycomb (from α-Li_2IrO_3) and H_2IrO_3-F (from β-Li_2IrO_3) can be another two possible structures for the reconstructed perovskite surface. Additionally, the reported IrOOH-brucite is considered even though its bulk activity is just comparable with the rutile IrO_2.(*49*) This is because, in previous studies on perovskite surface reconstruction, the reconstructed surfaces are also considered to be transition metal oxyhydroxides, which are active toward OER.

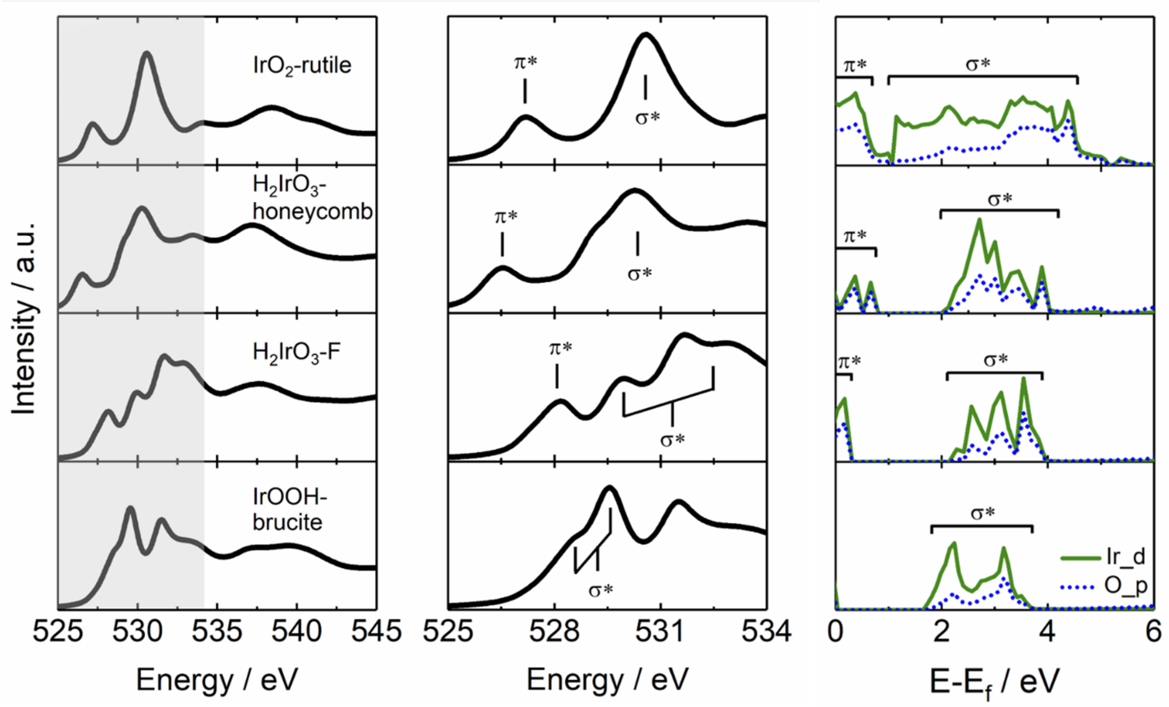

Fig. S22.

Simulated O K-edge spectra and electronic structures of possible structural motifs | The simulated O K-edge spectra (left column) from IrO_2-rutile, H_2IrO_3-honeycomb, H_2IrO_3-F, and IrOOH-brucite. The featured pre-edge peaks (middle column) are indexed with the corresponding PDOS (right column).

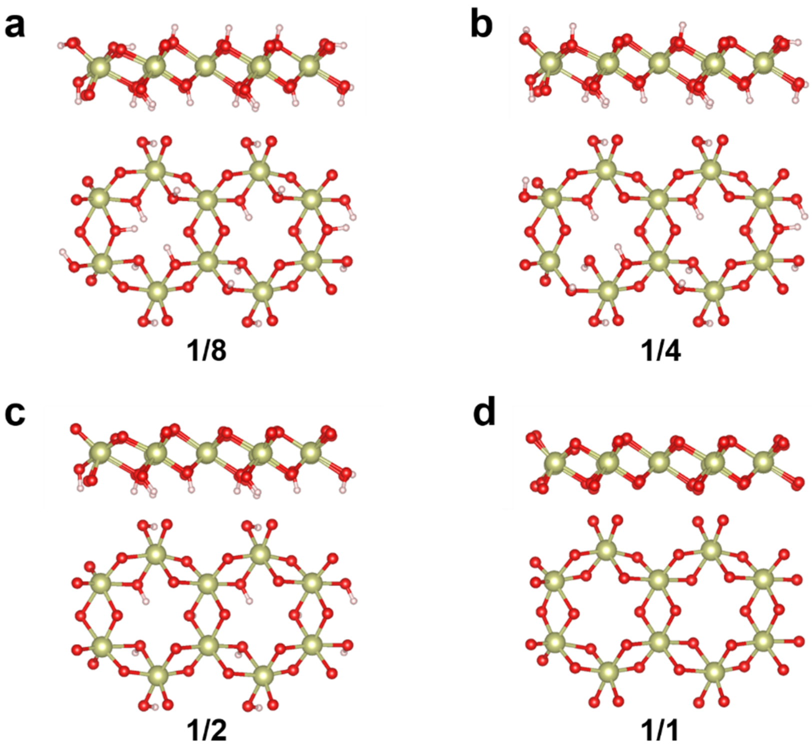

Fig. S23.

Deprotonation of the honeycomb H_2IrO_3 | The surface structures of the honeycomb H_2IrO_3 at different (from 1/8 to 1/1) deprotonation stages.

Table S1.

The calculated free energies (eV) of different surfaces.

		Initial state	IS1	IS2	IS3	IS4	Final state
$Sr(Sc_{0.5}Ir_{0.5})O_3$	No Sc vacancy	-825.83	-825.49	-822.80	-823.32	-823.84	-818.11
	With Sc vacancy	-812.30	-811.86	-810.29	-810.11	-810.69	-810.69
$Sr(Co_{0.5}Ir_{0.5})O_3$	No Co vacancy	-807.67	-807.21	-805.22	-805.49	/	805.86*
	With Co vacancy	-800.32	-800.12	-799.22	-798.37	-798.80	-791.87

*The Final state for $Sr(Co_{0.5}Ir_{0.5})O_3$ with no Co vacancy is a surface with a Sr adsorbed on the outer surface.

Table S2.
Refined structure information.

	$SrSc_{0.5}Ir_{0.5}O_3$			$SrCo_{0.5}Ir_{0.5}O_3$	
Space group	P *1 21/n 1*			I *1 2/m 1*	
a(Å)	5.6439(3)			5.5338(2)	
b(Å)	5.6407(3)			5.5477(1)	
c(Å)	7.9968(2)			7.8436(2)	
	Sr				
Wyckoff site	4i (0.487(7), 0.495(2), 0.232(0))			4i (0.504(1), 0, 0.249(0))	
Occ.	1			1	
U_{iso} ($Å^2$)	0.0142			0.0203	
	Sc			Co	
Wyckoff site	2c (0, 1/2, 0)	2d (1/2, 0, 0)		2a (0, 0, 0)	2d (0, 0, 1/2)
Occ.	0.887(1)	0.113(1)		0.860(3)	0.140(3)
U_{iso} ($Å^2$)	0.0261	0.0261		0.0361	0.0183
	Ir			Ir	
Wyckoff site	2c (0, 1/2, 0)	2d (1/2, 0, 0)		2a (0, 0, 0)	2d (0, 0, 1/2)
Occ.	0.113(1)	0.887(1)		0.140(3)	0.860(3)
U_{iso} ($Å^2$)	0.0261	0.0261		0.0361	0.0183
	O*				
Wyckoff site	4e (0.248(10), 0.265(9), -0.017(4))	4e (0.258(10), 0.738(10), -0.050(3))	4e (0.433(3), -0.009(11), 0.247(2))	4i (0.013(6), 0, 0.246(5))	8j (0.205(3), 0.282(3), 0.020(2))
Occ.	1				
U_{iso} ($Å^2$)	0.025				
	R_p=4.45%<br>R_{wp}=5.83%<br>Chi^2=1.720			R_p=3.22%<br>R_{wp}=4.07%<br>Chi^2=1.296	

*During the refinement, for O, the values of site occupancy and U_{iso} are constrained to be 1 and 0.025 $Å^2$, respectively.

Table S3.

Fitting parameters of the Fourier-transformed k^3-weighted Ir L_{III}-edge EXAFS. (k-range 3-12)

	**Ir-O (Å)**	**CN** *	**σ^2 ($Å^2$)** **	**ΔE_0** ***
SSI_pristine	1.953(0.006)	6.0(0.4)	0.0011(0.0008)	15.18(0.80)
SCI_pristine	1.950(0.007)	6.1(0.5)	0.0016(0.0009)	14.72(0.91)
SCI-H	1.973(0.006)	6.4(0.4)	0.0041(0.0009)	14.99(0.75)
IrO_2	1.983(0.005)	6.0(0.4)	0.0019(0.0006)	14.13(0.63)

*__CN:__ Coordination number
**__σ^2:__ Debye-Waller factor.
***__ΔE_0__: Energy shift

Table S4.

Fitting parameters of the XPS results from $SrSc_{0.5}Ir_{0.5}O_3$.

	**Sr***				**Sc**				**Ir**			
**As prepared**	$3d_{5/2}$	$3d_{3/2}$	$3d_{5/2}$	$3d_{3/2}$	$2p_{3/2}$	$2p_{1/2}$	$2p_{3/2}$	$2p_{1/2}$	$4f_{7/2}$	$4f_{5/2}$	satellite	
Peak position	132.6	134.4	133.1	134.9	400.7	405.3	402.0	406.5	63.9	66.9	69.5	/
FWHM	0.98	0.98	2.22	2.22	0.99	1.36	2.97	3.51	1.92	1.99	2.15	
**Cycled in KOH**	$3d_{5/2}$	$3d_{3/2}$	$3d_{5/2}$	$3d_{3/2}$	$2p_{3/2}$	$2p_{1/2}$	$2p_{3/2}$	$2p_{1/2}$	$4f_{7/2}$	$4f_{5/2}$	satellite	
Peak position	132.5	134.2	133.3	135.0	400.7	405.2	402.5	406.91	63.7	66.7	69.1	/
FWHM	1.03	1.03	2.42	2.42	1.08	1.54	2.11	2.36	2.13	2.04	1.85	
**Cycled in $HClO_4$**	$3d_{5/2}$	$3d_{3/2}$							$4f_{7/2}$	$4f_{5/2}$	$4f_{7/2}$	$4f_{5/2}$
Peak position	133.9	135.7	/				/		62.7	65.7	64.1	67.1
FWHM	2.52	2.72							1.48	1.69	3.11	2.83

*Considering the spin-orbit splitting, a relative area ratio of 2:3, 1:2, and 3:4 is considered for the doublets in Sr_3d, Sc (Co)_2p, and Ir_4f, separately. Spin-orbit splitting of 3 eV is considered for the doublets in Ir_4f. A Shirley background was applied during the fitting. All peaks are described as the convolution of Gaussian and Lorentzian functions.

Table S5.

Fitting parameters of the XPS results from $SrCo_{0.5}Ir_{0.5}O_3$.

	Sr				Co		Ir			
**As prepared**	$3d_{5/2}$	$3d_{3/2}$	$3d_{5/2}$	$3d_{3/2}$	$2p_{3/2}$	$2p_{1/2}$	$4f_{7/2}$	$4f_{5/2}$		
Peak position	132.0	133.7	132.9	134.7	780.6	795.9	63.1	66.2	/	
FWHM	0.82	0.82	2.5	2.5	2.68	2.68	2.21	2.42		
**Cycled in KOH**	$3d_{5/2}$	$3d_{3/2}$			$2p_{3/2}$	$2p_{1/2}$	$4f_{7/2}$	$4f_{5/2}$		
Peak position	133.1	134.7	/		780.6	795.6	62.9	66.0	/	
FWHM	2.78	2.45			3.15	3.15	2.41	2.31		
**Cycled in $HClO_4$**	$3d_{5/2}$	$3d_{3/2}$					$4f_{7/2}$	$4f_{5/2}$	$4f_{7/2}$	$4f_{5/2}$
Peak position	133.4	135.2	/		/		62.0	64.9	63.1	66.2
FWHM	1.98	1.98					1.45	1.43	2.74	2.90

Table S6.

The correction of zero-point energy and entropy of the adsorbed and gaseous species.

	ZPE(eV)	TS(eV)
*OOH	0.35	0
*O	0.05	0
*OH	0.31	0.01
H_2O	0.56	0.67
H_2	0.27	0.41

REFERENCES AND NOTES

1. J. Suntivich, K. J. May, H. A. Gasteiger, J. B. Goodenough, Y. Shao-Horn, A perovskite oxide optimized for oxygen evolution catalysis from molecular orbital principles. *Science* **334**, 1383–1385 (2011).

2. C. C. McCrory, S. Jung, J. C. Peters, T. F. Jaramillo, Benchmarking heterogeneous electrocatalysts for the oxygen evolution reaction. *J. Am. Chem. Soc.* **135**, 16977–16987 (2013).

3. C. Wei, Z. Feng, G. G. Scherer, J. Barber, Y. Shao-Horn, Z. J. Xu, Cations in octahedral sites: A descriptor for oxygen electrocatalysis on transition-metal spinels. *Adv. Mater.* **29**, 1606800 (2017).

4. L. C. Seitz, C. F. Dickens, K. Nishio, Y. Hikita, J. Montoya, A. Doyle, C. Kirk, A. Vojvodic, H. Y. Hwang, J. K. Norskov, T. F. Jaramillo, A highly active and stable $IrO_x/SrIrO_3$ catalyst for the oxygen evolution reaction. *Science* **353**, 1011–1014 (2016).

5. C. Liu, J. Qian, Y. Ye, H. Zhou, C.-J. Sun, C. Sheehan, Z. Zhang, G. Wan, Y.-S. Liu, J. Guo, S. Li, H. Shin, S. Hwang, T. Brent Gunnoe, W. A. Goddard III, S. Zhang, Oxygen evolution reaction over catalytic single-site Co in a well-defined brookite TiO_2 nanorod surface. *Nat. Catal.* **4**, 36–45 (2021).

6. C. Roy, B. Sebok, S. B. Scott, E. M. Fiordaliso, J. E. Sørensen, A. Bodin, D. B. Trimarco, C. D. Damsgaard, P. C. Vesborg, O. Hansen, I. E. L. Stephens, J. Kibsgaard, I. Chorkendorff, Impact of nanoparticle size and lattice oxygen on water oxidation on $NiFeO_xH_y$. *Nat. Catal.* **1**, 820–829 (2018).

7. T. Li, O. Kasian, S. Cherevko, S. Zhang, S. Geiger, C. Scheu, P. Felfer, D. Raabe, B. Gault, K. J. J. Mayrhofer, Atomic-scale insights into surface species of electrocatalysts in three dimensions. *Nat. Catal.* **1**, 300–305 (2018).

8. Jin, S. Are metal chalcogenides, nitrides, and phosphides oxygen evolution catalysts or bifunctional catalysts? *ACS Energy Lett.* **2**, 1937–1938 (2017).

9. H. N. Nong, T. Reier, H.-S. Oh, M. Gliech, P. Paciok, T. H. T. Vu, D. Teschner, M. Heggen, V. Petkov, R. Schlögl, T. Jones, P. Strasser, A unique oxygen ligand environment facilitates water oxidation in hole-doped $IrNiO_x$ core-shell electrocatalysts. *Nat. Catal.* **1**, 841–851 (2018).

10. A. Bergmann, E. Martinez-Moreno, D. Teschner, P. Chernev, M. Gliech, J. F. De Araújo, T. Reier, H. Dau, P. Strasser, Reversible amorphization and the catalytically active state of crystalline Co_3O_4 during oxygen evolution. *Nat. Commun.* **6**, 8625 (2015).

11. A. Bergmann, T. E. Jones, E. M. Moreno, D. Teschner, P. Chernev, M. Gliech, T. Reier, H. Dau, P. Strasser, Unified structural motifs of the catalytically active state of Co(oxyhydr)oxides during the electrochemical oxygen evolution reaction. *Nat. Catal.* **1**, 711–719 (2018).

12. E. Fabbri, M. Nachtegaal, T. Binninger, X. Cheng, B.-J. Kim, J. Durst, F. Bozza, T. Graule, R. Schäublin, L. Wiles, M. Pertoso, N. Danilovic, K. E. Ayers, T. J. Schmidt, Dynamic surface self-

reconstruction is the key of highly active perovskite nano-electrocatalysts for water splitting. *Nat. Mater.* **16**, 925–931 (2017).

13. Y. Chen, H. Li, J. Wang, Y. Du, S. Xi, Y. Sun, M. Sherburne, J. W. Ager, A. C. Fisher, Z. J. Xu, Exceptionally active iridium evolved from a pseudo-cubic perovskite for oxygen evolution in acid. *Nat. Commun.* **10**, 572 (2019).

14. K. J. May, C. E. Carlton, K. A. Stoerzinger, M. Risch, J. Suntivich, Y.-L. Lee, A. Grimaud, Y. Shao-Horn, Influence of oxygen evolution during water oxidation on the surface of perovskite oxide catalysts. *J. Phys. Chem. Lett.* **3**, 3264–3270 (2012).

15. M. Risch, A. Grimaud, K. J. May, K. A. Stoerzinger, T. J. Chen, A. N. Mansour, Y. Shao-Horn, Structural changes of cobalt-based perovskites upon water oxidation investigated by EXAFS. *J. Phys. Chem. C* **117**, 8628–8635 (2013).

16. C. W. Song, H. Suh, J. Bak, H. B. Bae, S.-Y. Chung, Dissolution-induced surface roughening and oxygen evolution electrocatalysis of alkaline-earth iridates in acid. *Chem* **5**, 3243–3259 (2019).

17. R. Zhang, N. Dubouis, M. Ben Osman, W. Yin, M. T. Sougrati, D. A. D. Corte, D. Giaume, A. Grimaud, A dissolution/precipitation equilibrium on the surface of iridium-based perovskites controls their activity as oxygen evolution reaction catalysts in acidic media. *Angew. Chem. Int. Ed.* **58**, 4571–4575 (2019).

18. R. Zhang, P. E. Pearce, Y. Duan, N. Dubouis, T. Marchandier, A. Grimaud, Importance of water structure and catalyst-electrolyte interface on the design of water splitting catalysts. *Chem. Mater.* **31**, 8248–8259 (2019).

19. A. Grimaud, K. J. May, C. E. Carlton, Y.-L. Lee, M. Risch, W. T. Hong, J. Zhou, Y. Shao-Horn, Double perovskites as a family of highly active catalysts for oxygen evolution in alkaline solution. *Nat. Commun.* **4**, 2439 (2013).

20. S. H. Chang, N. Danilovic, K.-C. Chang, R. Subbaraman, A. P. Paulikas, D. D. Fong, M. J. Highland, P. M. Baldo, V. R. Stamenkovic, J. W. Freeland, J. A. Eastman, N. M. Markovic, Functional links between stability and reactivity of strontium ruthenate single crystals during oxygen evolution. *Nat. Commun.* **5**, 4191 (2014).

21. B. M. Hunter, H. B. Gray, A. M. Muller, Earth-abundant heterogeneous water oxidation catalysts. *Chem. Rev.* **116**, 14120–14136 (2016).

22. W. T. Hong, M. Risch, K. A. Stoerzinger, A. Grimaud, J. Suntivich, Y. Shao-Horn, Toward the rational design of non-precious transition metal oxides for oxygen electrocatalysis. *Energ. Environ. Sci.* **8**, 1404–1427 (2015).

23. D. Chen, C. Chen, Z. M. Baiyee, Z. Shao, F. Ciucci, Nonstoichiometric oxides as low-cost and highly-efficient oxygen reduction/evolution catalysts for low-temperature electrochemical devices. *Chem. Rev.* **115**, 9869–9921 (2015).

24. T. Reier, Z. Pawolek, S. Cherevko, M. Bruns, T. Jones, D. Teschner, S. Selve, A. Bergmann, H. N. Nong, R. Schlögl, K. J. J. Mayrhofer, P. Strasser, Molecular insight in structure and activity of highly efficient, low-Ir Ir–Ni oxide catalysts for electrochemical water splitting (OER). *J. Am. Chem. Soc.* **137**, 13031–13040 (2015).

25. A. Grimaud, A. Demortière, M. Saubanère, W. Dachraoui, M. Duchamp, M.-L. Doublet, J.-M. Tarascon, Activation of surface oxygen sites on an iridium-based model catalyst for the oxygen evolution reaction. *Nat. Energy* **2**, 16189 (2016).

26. H. Li, Y. Chen, J. Ge, X. Liu, A. C. Fisher, M. P. Sherburne, J. W. Ager, Z. J. Xu, Active phase on $SrCo_{1-x}Fe_xO_{3-\delta}$ ($0\leq x\leq 0.5$) perovskite for water oxidation: Reconstructed surface versus remaining bulk. *JACS Au* **1**, 108–115 (2021).

27. P. P. Lopes, D. Y. Chung, X. Rui, H. Zheng, H. He, P. Farinazzo Bergamo Dias Martins, D. Strmcnik, V. R. Stamenkovic, P. Zapol, J. F. Mitchell, R. F. Klie, N. M. Markovic, Dynamically stable active sites from surface evolution of perovskite materials during the oxygen evolution reaction. *J. Am. Chem. Soc.* **143**, 2741–2750 (2021).

28. R. A. De Souza, M. S. Islam, E. Ivers-Tiffée, Formation and migration of cation defects in the perovskite oxide $LaMnO_3$. *J. Mater. Chem.* **9**, 1621–1627 (1999).

29. X. Rong, A. M. Kolpak, Ab initio approach for prediction of oxide surface structure, stoichiometry, and electrocatalytic activity in aqueous solution. *J. Phys. Chem. Lett.* **6**, 1785–1789 (2015).

30. P. Kayser, J. A. Alonso, F. J. Mompeán, M. Retuerto, M. Croft, A. Ignatov, M. T. Fernandez-Diaz, Crystal and magnetic structure of Sr_2BIrO_6 (B= Sc, Ti, Fe, Co, In) in the framework of multivalent iridium double perovskites. *Eur. J. Inorg. Chem.* **2015**, 5027–5038 (2015).

31. P. Kayser, B. J. Kennedy, B. Ranjbar, J. A. Kimpton, M. Avdeev, Spin-orbit coupling controlled ground state in the Ir(V) perovskites A_2ScIrO_6 (A = Ba or Sr). *Inorg. Chem.* **56**, 2204–2209 (2017).

32. J.-H. Choy, D.-K. Kim, S.-H. Hwang, G. Demazeau, D.-Y. Jung, XANES and EXAFS studies on the Ir-O bond covalency in ionic iridium perovskites. *J. Am. Chem. Soc.* **117**, 8557–8566 (1995).

33. S. Geiger, O. Kasian, M. Ledendecker, E. Pizzutilo, A. M. Mingers, W. T. Fu, O. Diaz-Morales, Z. Li, T. Oellers, L. Fruchter, A. Ludwig, K. J. J. Mayrhofer, M. T. M. Koper, S. Cherevko, The stability number as a metric for electrocatalyst stability benchmarking. *Nat. Catal.* **1**, 508–515 (2018).

34. R. Tang, Y. Nie, J. K. Kawasaki, D.-Y. Kuo, G. Petretto, G. Hautier, G.-M. Rignanese, K. M. Shen, D. G. Schlom, J. Suntivich, Oxygen evolution reaction electrocatalysis on $SrIrO_3$ grown using molecular beam epitaxy. *J. Mater. Chem. A* **4**, 6831–6836 (2016).

35. A. Minguzzi, O. Lugaresi, E. Achilli, C. Locatelli, A. Vertova, P. Ghigna, S. Rondinini, Observing the oxidation state turnover in heterogeneous iridium-based water oxidation catalysts. *Chem. Sci.* **5**, 3591–3597 (2014).

36. Z. Pavlovic, C. Ranjan, Q. Gao, M. van Gastel, R. Schlögl, Probing the structure of a water-oxidizing anodic iridium oxide catalyst using Raman spectroscopy. *ACS Catal.* **6**, 8098–8105 (2016).

37. P. Jovanovič, N. Hodnik, F. Ruiz-Zepeda, I. Arčon, B. Jozinović, M. Zorko, M. Bele, M. Šala, V. S. Šelih, S. Hočevar, M. Gaberšček, Electrochemical dissolution of iridium and iridium oxide particles in acidic media: Transmission electron microscopy, electrochemical flow cell coupled to inductively coupled plasma mass spectrometry, and X-ray absorption spectroscopy study. *J. Am. Chem. Soc.* **139**, 12837–12846 (2017).

38. E. Willinger, C. Massué, R. Schlögl, M. G. Willinger, Identifying key structural features of IrO_x water splitting catalysts. *J. Am. Chem. Soc.* **139**, 12093–12101 (2017).

39. C. Wei, S. Sun, D. Mandler, X. Wang, S. Z. Qiao, Z. J. Xu, Approaches for measuring the surface areas of metal oxide electrocatalysts for determining their intrinsic electrocatalytic activity. *Chem. Soc. Rev.* **48**, 2518–2534 (2019).

40. C. Wei, R. R. Rao, J. Peng, B. Huang, I. E. Stephens, M. Risch, Z. J. Xu, Y. Shao-Horn, Recommended practices and benchmark activity for hydrogen and oxygen electrocatalysis in water splitting and fuel cells. *Adv. Mater.* **31**, 1806296 (2019).

41. G. Li, L. Anderson, Y. Chen, M. Pan, P.-Y. A. Chuang, New insights into evaluating catalyst activity and stability for oxygen evolution reactions in alkaline media. *Sustain. Energy Fuels* **2**, 237–251 (2018).

42. F. Podjaski, D. Weber, S. Zhang, L. Diehl, R. Eger, V. Duppel, E. Alarcon-Llado, G. Richter, F. Haase, A. Fontcuberta i Morral, C. Scheu, B. V. Lotsch, Rational strain engineering in delafossite oxides for highly efficient hydrogen evolution catalysis in acidic media. *Nat. Catal.* **3**, 55–63 (2020).

43. D.-Y. Kuo, J. K. Kawasaki, J. N. Nelson, J. Kloppenburg, G. Hautier, K. M. Shen, D. G. Schlom, J. Suntivich, Influence of surface adsorption on the oxygen evolution reaction on IrO_2 (110). *J. Am. Chem. Soc.* **139**, 3473–3479 (2017).

44. H. N. Nong, L. J. Falling, A. Bergmann, M. Klingenhof, H. P. Tran, C. Spöri, R. Mom, J. Timoshenko, G. Zichittella, A. Knop-Gericke, S. Piccinin, J. Pérez-Ramírez, B. R. Cuenya, R.

Schlögl, P. Strasser, D. Teschner, T. E. Jones, Key role of chemistry versus bias in electrocatalytic oxygen evolution. *Nature* **587**, 408–413 (2020).

45. J. Hong, W. E. Gent, P. Xiao, K. Lim, D.-H. Seo, J. Wu, P. M. Csernica, C. J. Takacs, D. Nordlund, C.-J. Sun, K. H. Stone, D. Passarello, W. Yang, D. Prendergast, G. Ceder, M. F. Toney, W. C. Chueh, Metal–oxygen decoordination stabilizes anion redox in Li-rich oxides. *Nat. Mater.* **18**, 256–265 (2019).

46. M. Abbate, J. Goedkoop, F. De Groot, M. Grioni, J. Fuggle, S. Hofmann, H. Petersen, M. Sacchi, Probing depth of soft x-ray absorption spectroscopy measured in total-electron-yield mode. *Surf. Interface Anal.* **18**, 65–69 (1992).

47. L. Trotochaud, S. L. Young, J. K. Ranney, S. W. Boettcher, Nickel-iron oxyhydroxide oxygen-evolution electrocatalysts: The role of intentional and incidental iron incorporation. *J. Am. Chem. Soc.* **136**, 6744–6753 (2014).

48. F. Dionigi, P. Strasser, NiFe-based (oxy) hydroxide catalysts for oxygen evolution reaction in non-acidic electrolytes. *Adv. Energy Mater.* **6**, 1600621 (2016).

49. D. Weber, L. M. Schoop, D. Wurmbrand, S. Laha, F. Podjaski, V. Duppel, K. Müller, U. Starke, B. V. Lotsch, IrOOH nanosheets as acid stable electrocatalysts for the oxygen evolution reaction. *J. Mater. Chem. A* **6**, 21558–21566 (2018).

50. I. C. Man, H. Y. Su, F. Calle-Vallejo, H. A. Hansen, J. I. Martínez, N. G. Inoglu, J. Kitchin, T. F. Jaramillo, J. K. Nørskov, J. Rossmeisl, Universality in oxygen evolution electrocatalysis on oxide surfaces. *ChemCatChem* **3**, 1159–1165 (2011).

51. B. H. Toby, *EXPGUI*, a graphical user interface for *GSAS*. *J. Appl. Cryst.* **34**, 210–213 (2001).

52. O. Bunău, Y. Joly, Self-consistent aspects of x-ray absorption calculations. *J. Phys. Condens. Matter* **21**, 345501 (2009).

53. F. G. Sen, A. Kinaci, B. Narayanan, S. K. Gray, M. J. Davis, S. K. R. S. Sankaranarayanan, M. K. Y. Chan, Towards accurate prediction of catalytic activity in IrO_2 nanoclusters via first principles-based variable charge force field. *J. Mater. Chem. A* **3**, 18970–18982 (2015).

54. G. Kresse, J. Furthmüller, Efficient iterative schemes for ab initio total-energy calculations using a plane-wave basis set. *Phys. Rev. B. Condens. Matter* **54**, 11169–11186 (1996).

55. J. P. Perdew, K. Burke, M. Ernzerhof, Generalized gradient approximation made simple. *Phys. Rev. Lett.* **77**, 3865–3868 (1996).

56. S. L. Dudarev, G. A. Botton, S. Y. Savrasov, C. J. Humphreys, A. P. Sutton, Electron-energy-loss spectra and the structural stability of nickel oxide: An LSDA+U study. *Phys. Rev. B.* **57**, 1505–1509 (1998).

57. L. Wang, T. Maxisch, G. Ceder, Oxidation energies of transition metal oxides within the GGA+U framework. *Phys. Rev. B* **73**, 195107 (2006).

58. V. Stevanović, S. Lany, X. Zhang, A. Zunger, Correcting density functional theory for accurate predictions of compound enthalpies of formation: Fitted elemental-phase reference energies. *Phys. Rev. B* **85**, 115104 (2012).

59. H. J. Monkhorst, J. D. Pack, Special points for Brillouin-zone integrations. *Phys. Rev. B* **13**, 5188–5192 (1976).

60. J. K. Nørskov, J. Rossmeisl, A. Logadottir, L. Lindqvist, J. R. Kitchin, T. Bligaard, H. Jonsson, Origin of the overpotential for oxygen reduction at a fuel-cell cathode. *J. Phys. Chem. B* **108**, 17886–17892 (2004).

61. H. A. Hansen, J. Rossmeisl, J. K. Nørskov, Surface Pourbaix diagrams and oxygen reduction activity of Pt, Ag and Ni (111) surfaces studied by DFT. *Phys. Chem. Chem. Phys.* **10**, 3722–3730 (2008).

62. J. Rossmeisl, J. K. Nørskov, C. D. Taylor, M. J. Janik, M. Neurock, Calculated phase diagrams for the electrochemical oxidation and reduction of water over Pt(111). *J. Phys. Chem. B* **110**, 21833–21839 (2006).

63. A. Jain, S. P. Ong, G. Hautier, W. Chen, W. D. Richards, S. Dacek, S. Cholia, D. Gunter, D. Skinner, G. Ceder, K. A. Persson, Commentary: The Materials Project: A materials genome approach to accelerating materials innovation. *APL Mater.* **1**, 011002 (2013).

64. K. A. Persson, B. Waldwick, P. Lazic, G. Ceder, Prediction of solid-aqueous equilibria: Scheme to combine first-principles calculations of solids with experimental aqueous states. *Phys. Rev. B* **85**, 235438 (2012).

65. V. B. Parker, *Selected Values of Chemical Thermodynamic Properties: Tables for the Alkaline Earth Elements (Elements 92 Through 97 in the Standard Order of Arrangement)* (National Bureau of Standards, 1971), vol. 270.

66. M. Chassé, A. Juhin, D. Cabaret, S. Delhommaye, D. Vantelon, G. Calas, Influence of crystallographic environment on scandium K-edge X-ray absorption near-edge structure spectra. *Phys. Chem. Chem. Phys.* **20**, 23903–23912 (2018).

67. D. Weber, L. M. Schoop, D. Wurmbrand, J. Nuss, E. M. Seibel, F. F. Tafti, H. Ji, R. J. Cava, R. E. Dinnebier, B. V. Lotsch, Trivalent iridium oxides: Layered triangular lattice iridate $K_{0.75}Na_{0.25}IrO_2$ and oxyhydroxide IrOOH. *Chem. Mater.* **29**, 8338–8345 (2017).
